# Personalization and the Homogenization Debate in AI-Adaptive EFL Reading: Cognitive Load, Learner Agency, and Reading Development

**DOI:** 10.3390/bs16091478

**Published:** 2026-08-25

**Authors:** Latifah Hamdan Alghamdi, Talal Musaed Alghizzi

**Affiliations:** 1 English Language Center, King Khalid University, Abha 61421, Saudi Arabia; 2 College of Languages and Translation, Imam Mohammad Ibn Saud Islamic University (IMSIU), Riyadh 11564, Saudi Arabia; tmalghizzi@imamu.edu.sa

**Keywords:** AI-adaptive learning, cognitive load, perceived ownership of reading achievement, differentiated instruction, personalization–homogenization debate, construct interpretation, human-in-the-loop AI, assessment validity, adaptive educational systems

## Abstract

This exploratory cluster-randomized mixed-methods study examined reading development and learner experience across AI-adaptive, teacher-differentiated, and non-differentiated EFL reading instruction. Eighty-seven university-level EFL learners, classified by CEFR proficiency, completed a 12-week intervention across four intact classrooms: two AI-adaptive, one teacher-differentiated, and one non-differentiated. Reading development was assessed using equated parallel-form IELTS-format tests calibrated through Rasch modeling and CEFR cut-score mapping. Learner experience was assessed using multi-item measures with satisfactory internal consistency and preliminary evidence of structural validity for cognitive load, engagement, instructional comfort, and perceived ownership of reading achievement, supplemented by qualitative reflections. Because instructional condition was assigned at the classroom level and only four clusters were available, reading development patterns were interpreted primarily through descriptive comparison of the recoverable model-implied pre-to-post patterns, with participant-level mixed-design ANOVA and cluster-adjusted mixed-effects modeling reported as secondary analyses. The pooled AI-adaptive classrooms showed a larger model-implied pre-to-post increase than the comparison classrooms, while both secondary analyses showed the same directional pattern but substantial uncertainty. Descriptively, learners in the AI-adaptive classrooms reported lower cognitive load and higher engagement, instructional comfort, and perceived ownership of reading achievement. These findings provide preliminary classroom-based evidence of favorable patterns associated with AI-adaptive personalization but do not establish causal or generalizable instructional effects. Larger cluster-randomized studies are needed to separate instructional effects from classroom-level influences and examine proficiency-related differences.

## 1. Introduction

The rapid integration of artificial intelligence (AI) into language education has fundamentally reshaped how instructional input is delivered, sequenced, and experienced. In English as a Foreign Language (EFL) reading, AI-adaptive systems now promise finely calibrated personalization through real-time analysis of learner performance, enabling dynamic adjustments to text difficulty, lexical density, cohesion, and scaffolding intensity (Holmes et al., 2019; Zawacki-Richter et al., 2019). These developments position AI not merely as a supplementary tool but as an active mediator of learning pathways, cognitive effort, and learner experience.

Personalization has long been regarded as a pedagogical ideal in reading instruction. Research in applied linguistics and educational psychology suggests that comprehension improves when instructional input aligns with learners’ proficiency levels and cognitive capacities, reducing unnecessary cognitive load while supporting meaningful engagement (Grabe & Stoller, 2019; Nation, 2013; Sweller et al., 2019). AI-adaptive systems may offer new possibilities for implementing this principle at scale by providing responsive instructional adjustments within heterogeneous classrooms. However, emerging scholarship has raised questions about whether algorithmically mediated personalization could also shape learner autonomy, agency, and engagement patterns in unintended ways (Bayne, 2015; Selwyn, 2019).

From this perspective, personalization may coexist with subtle forms of instructional standardization, as optimization processes can influence both cognitive effort and learners’ sense of ownership over learning. These concerns are particularly relevant in EFL reading (Kintsch, 1998), where instructional regulation of text complexity and cohesion can substantially affect cognitive load and engagement (Sweller et al., 2019). At the same time, some scholars have suggested that extensive automation may encourage greater reliance on external support systems, potentially influencing strategic self-regulation and cognitive engagement (Risko & Gilbert, 2016). Although these broader debates provide theoretical motivation for the present study, the empirical focus is more narrowly centered on comparative differences in reading development, cognitive load, engagement, instructional comfort, and self-reported perceived ownership of reading achievement across instructional conditions. Accordingly, this study does not directly operationalize homogenization as a behavioral construct; instead, it examines learner outcomes and experiences that may inform broader discussions of AI-mediated personalization.

Beyond cognition, AI-mediated instruction intersects with learner identity and agency. Research on AI-assisted writing shows that learners may experience both empowerment and alienation, depending on the degree of control they perceive over the learning process (Carless & Boud, 2018; Gabay et al., 2026). Comparable identity-related dynamics in AI-adaptive reading remain largely unexplored, even though reading is a central site of meaning-making, autonomy development, and academic self-concept formation, especially in EFL contexts (Norton, 2013). Crucially, most existing studies examine AI-based reading tools in isolation or only in contrast to traditional instruction. Far fewer studies compare AI-adaptive personalization with expert human-designed differentiated instruction, a comparison that is pedagogically and theoretically essential. Without this benchmark, it remains unclear whether AI is associated with outcomes that differ from those observed under expert human differentiation. Against this backdrop, the present study investigates differences in learning outcomes, cognitive load, engagement, and perceived ownership of reading achievement across instructional conditions.

Regardless of reading development, the present study contributes to emerging discussions on interpreting constructs in AI-enabled learning environments. Specifically, this study examines how AI-adaptive support is associated with key psychological constructs, including cognitive load, engagement, instructional comfort, and learner agency, and compares these patterns with those observed under teacher-differentiated instruction. In this sense, this study addresses not only instructional outcomes but also how learner experience constructs are interpreted within adaptive educational systems.

### 1.1. Purpose and Objective of Study

The primary purpose of this study is to examine differences among three instructional conditions: AI-adaptive instruction, teacher-differentiated instruction, and traditional non-differentiated instruction. More specifically, this study compares two distinct adaptive instructional architectures: continuous algorithmically mediated personalization and conventional teacher-differentiated instruction. This study therefore examines whether learner outcomes and experiences differ across these instructional architectures and investigates associated patterns in reading development, perceived cognitive load, engagement, instructional comfort, and perceived ownership of reading achievement. To achieve this purpose, this study is guided by the following objectives:

1. To describe the recoverable pre-to-post reading development patterns across the participating classroom sections.
2. To examine differences in cognitive and affective outcomes across these instructional approaches by analyzing differences in learners’ perceived cognitive load, engagement, and instructional comfort.
3. To investigate learners’ perceived ownership of their reading achievement and agency, focusing on how learners’ perceptions of ownership of reading achievement differed across instructional conditions.

### 1.2. Gap in Knowledge and Research Questions

Despite rapid growth in AI-mediated language learning research, several critical gaps remain. First, most AI-based reading studies prioritize outcome measures while neglecting underlying cognitive and affective mechanisms. Learners’ cognitive load, emotional comfort, and engagement, central determinants of sustainable reading development, are rarely examined alongside performance gains (Zawacki-Richter et al., 2019). Second, empirical comparisons between AI-adaptive personalization and expert human differentiation are notably scarce. Existing studies typically contrast AI-supported instruction with undifferentiated instruction, leaving open how outcomes associated with continuous AI-adaptive support compare with those associated with established teacher-differentiated instruction practices (Tomlinson, 2014).

Third, learner identity and authorship remain marginal in reading research. While concerns about agency and cognitive offloading have gained attention in AI-assisted writing, parallel investigations in AI-adaptive reading are virtually absent, despite reading’s foundational role in academic identity construction (Norton, 2013; Risko & Gilbert, 2016). Finally, proficiency-level sensitivity is under-theorized. Mixed findings suggest that AI personalization may benefit some learners while constraining others, yet few studies employ stratified designs that can detect differential effects across proficiency bands. By addressing these gaps through a comparatively rigorous classroom-based cluster-randomized comparative design, the present study contributes to the ongoing personalization–homogenization debate and advances understanding of how cognitive load, perceived ownership of reading achievement, and learning pathways differ across AI-mediated instructional conditions in EFL reading.

To achieve the above objectives and address the identified knowledge gaps, this study is guided by the following research questions:

RQ1. What recoverable pre-to-post reading development patterns were observed across the participating classroom sections?
RQ2. How do learners’ perceived cognitive load, engagement, and instructional comfort differ across AI-adaptive, teacher-differentiated, and non-differentiated instructional conditions?
RQ3. How do perceptions of ownership of reading achievement differ across AI-adaptive, teacher-differentiated, and non-differentiated instructional conditions?

## 2. Literature Review

### 2.1. AI-Adaptive Reading Systems in Language Education

Artificial intelligence has expanded possibilities for individualized instruction by enabling systems that diagnose learner performance, adjust task difficulty, and provide real-time scaffolding. AI-adaptive learning models integrate learner analytics, natural language processing, and machine learning to dynamically tailor instructional pathways (Holmes et al., 2019). In reading instruction, such systems can modulate text length, lexical frequency, syntactic complexity, and cohesion, aligning input with learners’ evolving proficiency. Empirical research suggests that AI-driven personalization can enhance reading comprehension by reducing mismatches between task difficulty and learner ability (Kukulska-Hulme & Viberg, 2018; Hilali et al., 2023; Li et al., 2014). Adaptive platforms such as Reading Plus and iReady sequence texts based on learners’ response patterns and have demonstrated positive effects on comprehension outcomes (Gevorgyan, 2024). Despite these affordances, existing research has largely examined AI personalization in isolation.

### 2.2. Human Differentiation and Tiered Reading Instruction

Differentiated instruction is a foundational pedagogical principle emphasizing adjustments to content, process, and tasks to accommodate learner diversity (Tomlinson, 2014). In reading instruction, differentiation commonly involves tiered tasks, leveled texts, flexible grouping, and scaffolded comprehension activities, which can enhance engagement and comprehension when implemented effectively (Hall et al., 2002). However, human-designed differentiation faces substantial practical constraints (Santangelo & Tomlinson, 2012; VanLehn, 2011).

### 2.3. Reading Comprehension and Cognitive Load

Reading comprehension is a cognitively complex process involving lexical access, syntactic parsing, inference generation, and integration of textual information (Kintsch, 1998). Cognitive Load Theory posits that learning is optimized when instructional materials align with working memory capacity, thereby regulating learners’ cognitive demands and supporting schema construction (Sweller et al., 2019; Nation, 2013; Grabe & Stoller, 2019; Ritter et al., 2007).

### 2.4. Engagement, Emotional Comfort, and Learner Experience

Learner engagement strongly predicts reading achievement and academic persistence (Fredricks et al., 2004). AI-based systems often claim to enhance engagement through personalized pacing, immediate feedback, and interactive task formats (Alghamdi & Alghizzi, 2026; Zawacki-Richter et al., 2019). Some studies report increased motivation when learners perceive tasks as optimally challenging rather than frustrating or monotonous (Pavan, 2020). However, engagement is not solely behavioral. Emotional comfort, encompassing anxiety levels, clarity of task demands, and perceived control, is equally critical for sustained comprehension. Research in computer-assisted language learning suggests that opaque algorithms or unfamiliar interfaces may provoke anxiety, particularly among lower-proficiency learners (Alsaawi et al., 2025).

### 2.5. Perceived Ownership of Reading Achievement and Proficiency-Level Differences

Perceived ownership of reading achievement, the perception that academic achievement reflects one’s own effort and agency, is central to literacy development. In the present study, this construct is assessed through a four-item subscale and is used in a restricted operational sense: the items index perceived ownership of reading achievement, attribution of success to personal effort, and the belief that instructional support did not replace the learner’s own thinking, rather than the broader sociocultural or identity-based sense of authorship sometimes used elsewhere in the literature; general claims about learner identity beyond this operational scope are not intended.

Studies in AI-assisted writing indicate mixed effects, with some learners reporting enhanced confidence and others experiencing diminished ownership due to machine-generated support (Carless & Boud, 2018; Gabay et al., 2026). Comparable concerns apply to reading: overly aggressive adaptivity may undermine learners’ sense of authentic achievement, particularly in EFL contexts where identity construction is fragile (Norton, 2013; Grabe & Stoller, 2019; Xie et al., 2019; De Lima, 2022; Zheng & Yu, 2019).

### 2.6. Theoretical Framework

#### AI Personalization in L2 Reading

Personalization has long been recognized as a foundational principle in second language (L2) pedagogy, reflecting systematic differences among learners in proficiency, background knowledge, cognitive resources, and strategic repertoires (Dörnyei & Ryan, 2015; Ellis, 2015). In L2 reading instruction, personalization has traditionally been enacted through teacher-led differentiation, scaffolded materials, and sequenced task design (Grabe & Stoller, 2019; Nation, 2013). The emergence of artificial intelligence (AI) extends these practices by enabling data-driven, adaptive personalization that dynamically adjusts textual complexity, lexical support, and pacing in response to learner performance (Kukulska-Hulme et al., 2022; Zawacki-Richter et al., 2019).

In AI-supported L2 reading, personalization is typically operationalized through adaptive modulation of input difficulty, lexical assistance, and syntactic complexity, drawing on principles of comprehensible input, interaction, and developmental readiness (Ellis, 2017; Long, 2015; Crossley et al., 2018; Ma et al., 2014).

### 2.7. Cognitive Load, Agency, and Identity in L2 Reading

Cognitive Load Theory provides a robust framework for understanding how instructional design influences learning by regulating demands on working memory (Sweller et al., 2019). In L2 reading, intrinsic cognitive load is shaped by linguistic complexity and discourse structure, while extraneous load arises from poorly aligned instructional supports (Paas et al., 2003; Leppink et al., 2013). Effective instructional design seeks to optimize these load components to facilitate germane processing and learning. AI-adaptive reading systems are frequently justified on cognitive grounds because they are intended to regulate instructional demands by calibrating input to learners’ proficiency levels. Although such systems are often theorized to reduce unnecessary cognitive demands, the present study measured only learners’ overall perceived cognitive load and therefore does not distinguish among intrinsic, extraneous, and germane cognitive load (Plass & Pawar, 2020). However, reading is not solely a cognitive activity; it is also an agentive and identity-related practice (Grabe & Stoller, 2019; Afflerbach et al., 2015; Norton, 2013; Mercer, 2014).

### 2.8. The Personalization–Homogenization Debate

Although personalization is widely assumed to promote individualization, AI-adaptive systems introduce a paradox: individualized adaptation may produce homogenized learning trajectories. The personalization–homogenization debate concerns the possibility that algorithmically personalized instruction may inadvertently encourage greater similarity in learning experiences, decision processes, or instructional pathways (Williamson & Eynon, 2020; Selwyn, 2019). The present study does not operationalize or directly measure homogenization. Instead, the concept provides a theoretical lens for interpreting learner outcomes and experiences associated with AI-adaptive instruction.

### 2.9. The Cognitive Alignment Model of AI-Adaptive Reading (CAM-AIR)

**Figure 1:**
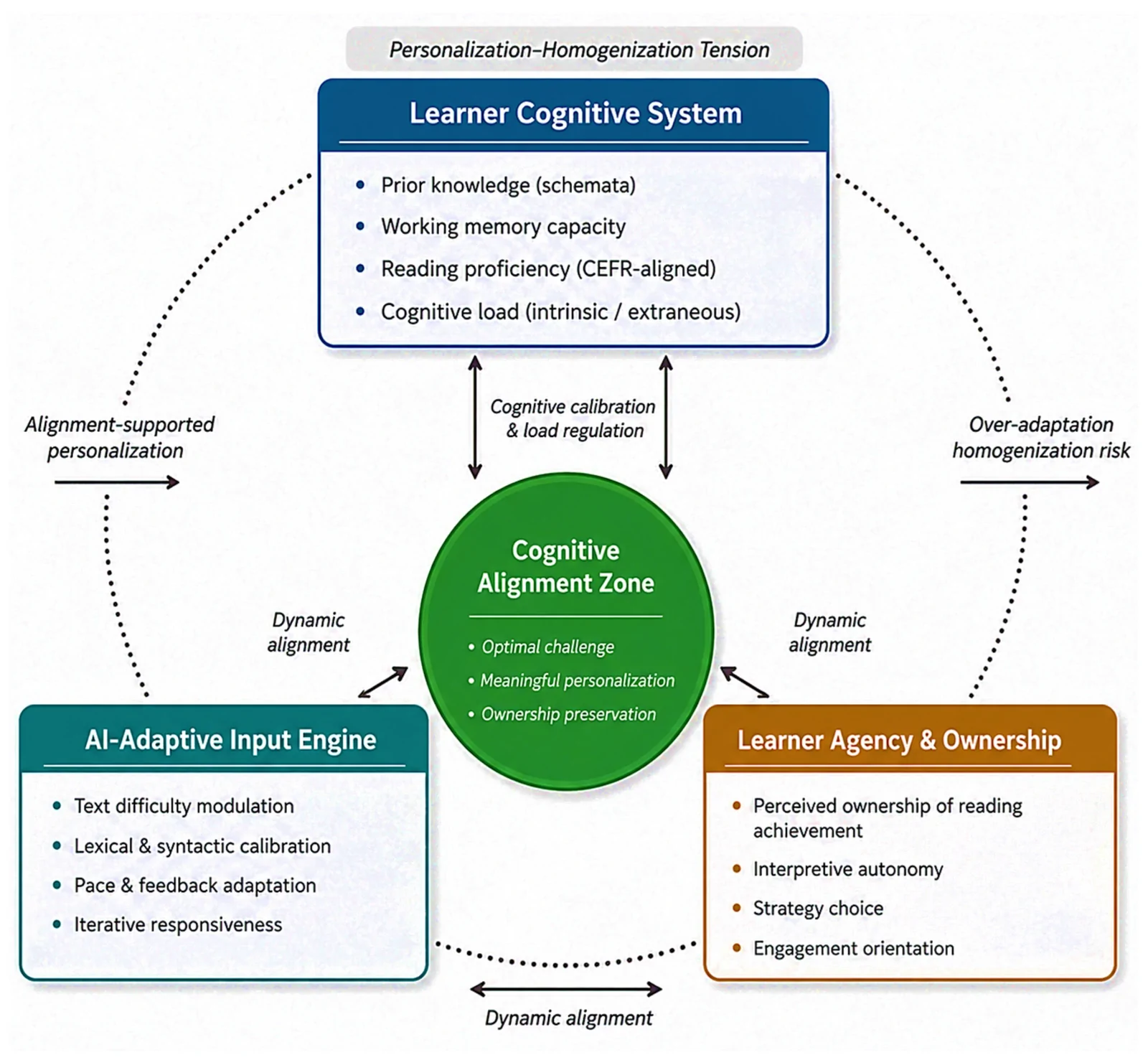
Conceptual representation of the Cognitive Alignment Model of AI-Adaptive Reading (CAM-AIR).

This study proposes the Cognitive Alignment Model of AI-Adaptive Reading (CAM-AIR) as a preliminary interpretive framework for organizing and explaining the observed relationships among adaptive instructional support, learner experience, and reading development. CAM-AIR is intended to generate theoretically informed hypotheses rather than to represent an empirically validated explanatory model (Figure 1). Drawing on Cognitive Load Theory, sociocognitive perspectives on agency, and adaptive learning research, CAM-AIR posits that effective personalization occurs within a Cognitive Alignment Zone, where instructional adaptation supports processing efficiency while preserving interpretive autonomy and learners’ perceived ownership of their reading achievement (Sweller et al., 2019; Lantolf & Thorne, 2006; Luckin et al., 2016). By shifting attention from adaptation intensity to alignment quality, the model provides a principled framework for explaining when AI personalization facilitates learning and when it may reduce agency or erode learners’ perceived ownership of their reading achievement. Importantly, CAM-AIR is advanced as a preliminary interpretive framework, informed by the present findings, rather than as a fully validated predictive model.

From this perspective, CAM-AIR may also be viewed as a construct interpretation framework that links adaptive instructional processes to learner experience constructs. Rather than serving as a predictive model, the framework provides a conceptual structure for interpreting relationships among cognitive load, engagement, agency, and adaptive instructional support in AI-enabled learning environments.

The present study provides only partial empirical support for elements of CAM-AIR. Reading development, perceived cognitive load, engagement, instructional comfort, and perceived ownership of reading achievement were measured directly. In contrast, ownership preservation and homogenization risk were not directly operationalized and are therefore inferred conceptually rather than empirically demonstrated. Accordingly, CAM-AIR should be interpreted as a hypothesis-generating framework that organizes the present findings while identifying relationships requiring direct empirical evaluation in future research.

## 3. Methodology

### 3.1. Research Design and Analytical Framework

This study employed a cluster-randomized comparative classroom intervention in which intact English for Academic Purposes (EAP) class sections were randomly assigned to one of three instructional conditions: (a) AI-adaptive instruction, (b) teacher-differentiated instruction, and (c) non-differentiated instruction. Cluster randomization was required because students were enrolled in fixed class sections that could not be reorganized. Four intact sections participated (N = 87): two were allocated to the AI-adaptive condition (*N* = 44), one to the teacher-differentiated condition (*N* = 21), and one to the non-differentiated condition (*N* = 22). All groups were taught by the same instructor using scripted lesson plans and condition-specific protocols to minimize instructor effects and cross-condition contamination.

Because only four classroom clusters were available, this study provides exploratory classroom-level evidence rather than definitive causal estimates. Moreover, each teacher-differentiated and non-differentiated condition was represented by a single cluster, leaving instructional condition partially confounded with cluster membership. Consequently, observed differences may reflect both instructional characteristics and unmeasured class-level factors (e.g., classroom climate, peer composition, group dynamics, or motivation). Although the sections were broadly comparable in demographic characteristics and CEFR distributions, baseline equivalence was not established on the continuous reading measure, and residual cluster-specific influences cannot be excluded. To assess the robustness and clustering sensitivity of the observed patterns, mixed-effects modeling, intraclass correlation estimation, design effect adjustment, and sensitivity analyses were conducted. These procedures improve transparency but cannot overcome the limited number and unequal allocation of clusters or separate instructional condition from cluster-specific influences.

#### Cluster Randomization Procedure

Randomization was conducted at the classroom level because institutional scheduling precluded assigning students individually. Before baseline assessment, a research assistant not involved in instruction or outcome assessment assigned anonymous identification codes to the four intact EAP sections. A computer-generated random-number sequence then allocated the sections to the three instructional conditions, resulting in two AI-adaptive classes and one class each in the teacher-differentiated and non-differentiated conditions. Given the availability of only four clusters, stratified or constrained randomization was not feasible.

Allocation occurred before the intervention and prior to baseline instructional activities. Because of the nature of the intervention, allocation concealment was not possible; the instructor needed to know group assignments to prepare condition-specific materials. Nevertheless, baseline assessments were administered under standardized conditions, and scoring, psychometric calibration, and statistical analyses followed prespecified protocols to minimize investigator bias.

### 3.2. Participants and Instructional Context

Participants were 87 female first-year EFL learners (18–21 years) enrolled in the English Language Center, College of Languages and Translation, Imam Mohammad Ibn Saud Islamic University, Saudi Arabia. All had completed approximately 6–8 years of formal English instruction and were following an institutional curriculum targeting CEFR A2–B1 reading proficiency. Although enrolled in the same curricular level, diagnostic assessment revealed substantial variability in reading proficiency, justifying proficiency stratification and reinforcing the relevance of comparing adaptive and differentiated instruction (Table 1).

The single-gender sample reflects the institutional teaching context. Although this limits generalizability, it enhances internal validity by controlling for gender-related instructional and interactional variation. Participation was voluntary, and ethical approval was obtained in accordance with institutional research guidelines. All participants were provided informed consent after receiving information about the study’s objectives, procedures, confidentiality protections, and assurance that participation would not affect course grades or academic standing.

**Figure 2:**
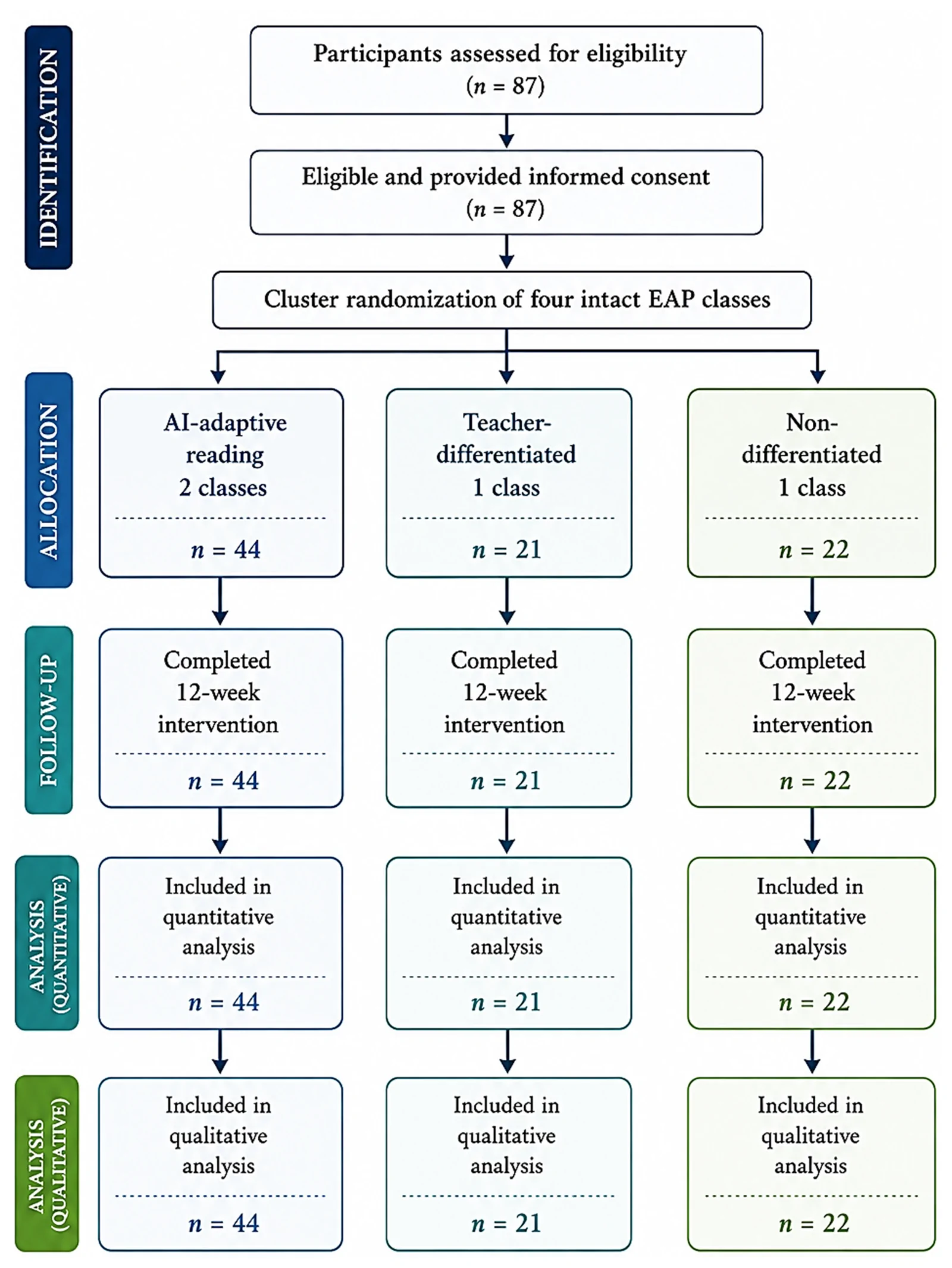
Participant Recruitment, Allocation, Follow-Up, and Analysis Flow.

Figure 2 summarizes participant recruitment, classroom allocation, intervention completion, and inclusion in the quantitative and qualitative analyses. No participants withdrew after enrollment, and all 87 participants completed the intervention and post-intervention assessments.

### 3.3. Proficiency Stratification and Group Assignment

Prior to the intervention, all participants completed the CEFR-aligned reading comprehension pre-test described in Section 3.7. The assessment served two purposes: (a) to classify learners into A2 and B1 proficiency bands for descriptive and analytical purposes, and (b) to evaluate baseline equivalence across class sections. Because this study employed a cluster-randomized design, students remained in their intact class sections following randomization. Instead, the proficiency composition of each class was examined to confirm broadly comparable proportions of A2- and B1-level learners across instructional conditions. Minor imbalances were addressed statistically through baseline-equivalence testing and covariate-adjusted analyses rather than individual reassignment.

Table 2 summarizes the categorical CEFR band (A2/B1) distribution across instructional conditions. Although the proportions of A2- and B1-level learners were broadly comparable, the model-based baseline estimates reported in Section 4.1 indicated an initial difference on the continuous Rasch-scaled reading measure between the AI-adaptive and non-differentiated conditions. Accordingly, similarity in CEFR band distributions was not interpreted as evidence of equivalence on the continuous reading measure. Longitudinal analyses therefore explicitly modeled baseline differences using the Time × Condition interaction and a baseline-adjusted ANCOVA sensitivity analysis.

### 3.4. Instructional Materials and Content Control

All instructional conditions addressed the same reading topics, learning objectives, and underlying propositional content to ensure curricular comparability. Materials were drawn from a common bank of CEFR-aligned passages based on the institutional curriculum. While content remained constant, its linguistic realization varied by instructional architecture. In the AI-adaptive condition, lexical support, syntactic complexity, scaffolding, and question difficulty were adjusted dynamically. Comparable adaptations were implemented through teacher-designed tiered materials in the differentiated condition, whereas the non-differentiated condition used a single unmodified version of each passage.

To preserve internal validity, the following elements were held constant across conditions:

- Reading topics and thematic content.
- Total instructional time and session length.
- Assessment tasks and evaluation criteria.

The principal source of variation was the instructional architecture, including adaptation source, responsiveness, continuity, feedback density, and personalization granularity (Section 3.5.1). This design minimized content-related confounding and improved curricular comparability across conditions; however, observed differences cannot be attributed uniquely to instructional condition because classroom-level influences remain inseparable from condition in the four-cluster design.

### 3.5. Instructional Conditions

#### 3.5.1. AI-Adaptive Personalized Reading Instruction (G1)

The AI-adaptive condition preserved the propositional meaning and learning objectives of each reading passage while dynamically modifying selected linguistic and instructional features according to learner performance. Rather than assigning learners to fixed proficiency groups, a researcher-configured adaptive platform continuously calibrated text complexity, lexical support, syntactic complexity, scaffolding, comprehension-question difficulty, and feedback specificity to align instructional demands with learners’ evolving reading ability.

The platform employed a transparent, deterministic, rule-based adaptation engine rather than machine learning or generative AI. Although the platform employed deterministic rule-based adaptation rather than machine learning, it is referred to as an AI-adaptive system because it continuously analyzed learner performance and autonomously selected instructional responses without instructor intervention, consistent with the operational definition of AI-adaptive instruction adopted in this study. It continuously monitored response accuracy, response latency, and error patterns, then applied predefined CEFR-aligned decision rules informed by Cognitive Load Theory. For example, sustained high performance (≥80% accuracy) triggered progression to more inferential tasks with reduced scaffolding, whereas repeated errors or prolonged response times prompted simplified syntax, additional lexical support, and more explicit guidance. Adaptation was therefore performance-responsive rather than predictive, ensuring transparency, instructional consistency, and reproducibility. Reading passages were selected from a researcher-developed, pre-reviewed CEFR-aligned text bank. The system did not generate or rewrite texts; instead, it selected appropriate passages and adjusted scaffolding, lexical support, question difficulty, and instructional prompts. All materials were reviewed before implementation to ensure semantic equivalence, curricular alignment, and consistency of learning objectives.

In addition, the platform recorded learner identification codes, response accuracy, response latency, adaptation levels, completed activities, and instructional progression. No personally identifiable information was transmitted to external providers. Appendix A provides technical specifications of the adaptive architecture, decision rules, and logging procedures. This comparison therefore examined two adaptive instructional architectures rather than AI versus non-AI instruction. The AI-adaptive condition implemented continuous algorithm-mediated personalization, whereas teacher differentiation relied on intermittent instructor-mediated adaptation constrained by classroom pacing and teacher attention. Because these architectures differed simultaneously in adaptation source, responsiveness, continuity, feedback density, and personalization granularity, the study was not designed to isolate the independent effect of AI. Accordingly, the findings should be interpreted as reflecting the combined characteristics of the instructional systems rather than AI alone.

#### 3.5.2. Teacher-Designed Differentiated Reading Instruction (G2)

In the teacher-differentiated condition, instruction was delivered through pedagogically grounded, teacher-created tiered tasks aligned with learners’ proficiency levels. All learners worked with the same reading texts to ensure content equivalence; however, task demands and the depth of scaffolding varied by proficiency level. Differentiation strategies included the following:

(a) Tiered comprehension questions (literal vs. inferential focus);
(b) Adjusted scaffolding prompts (explicit guidance vs. strategic hints);
(c) Variable depth of inferential and critical reading tasks.

This condition reflects established practices of differentiation in EFL pedagogy, drawing on teachers’ professional judgment, contextual awareness, and instructional experience.

#### 3.5.3. Traditional Non-Differentiated Reading Instruction (G3)

The control group followed the standard institutional reading curriculum without adaptive or differentiated mechanisms. All learners received identical texts, tasks, and instructional support, regardless of proficiency level. Instruction consisted of uniform pre-reading explanation, guided reading, and whole-class review of comprehension tasks. (see Table 3). No systematic adjustments were made to task difficulty, scaffolding intensity, or feedback specificity.

### 3.6. Study Procedure and Treatment Implementation

The instructional intervention was implemented over twelve weeks following a standardized, phase-based protocol designed to ensure treatment fidelity, comparability across groups, and replicability.

Phase 1: Orientation and Pre-Intervention Assessment (Week 1)

Phase 1 spanned two 90-min sessions and focused on orientation, ethical briefing, and baseline assessment. During the orientation session, participants were informed of the study’s objectives, procedures, and assessment schedule. Ethical assurances, including voluntary participation, confidentiality, and non-impact on course grades, were clearly communicated. Learners also received a brief orientation to the instructional format specific to their assigned condition, without disclosure of comparative hypotheses to prevent expectancy effects.

In the pre-test session, all participants completed a CEFR-aligned reading comprehension test under standardized examination conditions. The assessment measured literal comprehension, inferential understanding, and the construction of interpretive meaning. Pre-test scores were used for proficiency stratification, baseline assessment, and the longitudinal pre-to-post analyses; they were not used for evaluative grading.

Phase 2: Instructional Treatment (Weeks 2–11)

The instructional treatment phase consisted of three 60-min sessions per week, totaling 3 h per group. All groups covered the same reading topics and texts during this phase. To control for extraneous instructional variables, instructional time was identical across groups, the same instructor taught all conditions, and assessment expectations were held constant. The study compared two adaptive instructional architectures and one non-adaptive instructional architecture rather than isolating a single AI component (see Table 4).

*Qualitative Sample and Data Management*

Open-ended reflections were obtained from all 87 participants immediately after completing the instructional intervention. Responses were collected across all instructional conditions (AI-adaptive: *N* = 44; teacher-differentiated: *N* = 21; non-differentiated: N = 22). Participants completed the reflections anonymously using identification codes assigned for research purposes. These identifiers are used when presenting illustrative quotations to preserve participant confidentiality while allowing readers to identify the instructional condition from which each quotation originated.

Phase 3: Post-Intervention Assessment (Week 12)

Participants completed a learner perception questionnaire assessing cognitive load, engagement, instructional comfort, and perceived ownership of reading achievement, followed by open-ended written reflections on their learning experiences, perceived benefits, challenges, and sense of agency. Participants completed the reflections immediately after the intervention under classroom conditions and wrote them in English as part of their regular English for Academic Purposes coursework; therefore, no translation was required. Qualitative data were analyzed using Braun and Clarke’s (2006) six-phase inductive thematic analysis. Analysis involved repeated familiarization with the data, inductive coding, theme development and refinement, and selection of representative quotations. No predetermined codebook was used. Additionally, both authors independently familiarized themselves with the data and developed preliminary inductive codes. The authors then discussed the resulting interpretations to refine theme definitions and ensure consistent application of the analytical framework. Given the exploratory purpose of the qualitative component, the analysis aimed to identify recurring patterns of meaning rather than quantify coding agreement or estimate inter-coder reliability.

#### 3.6.1. Structure of Each Instructional Session (60 Minutes)

**Figure 3:**
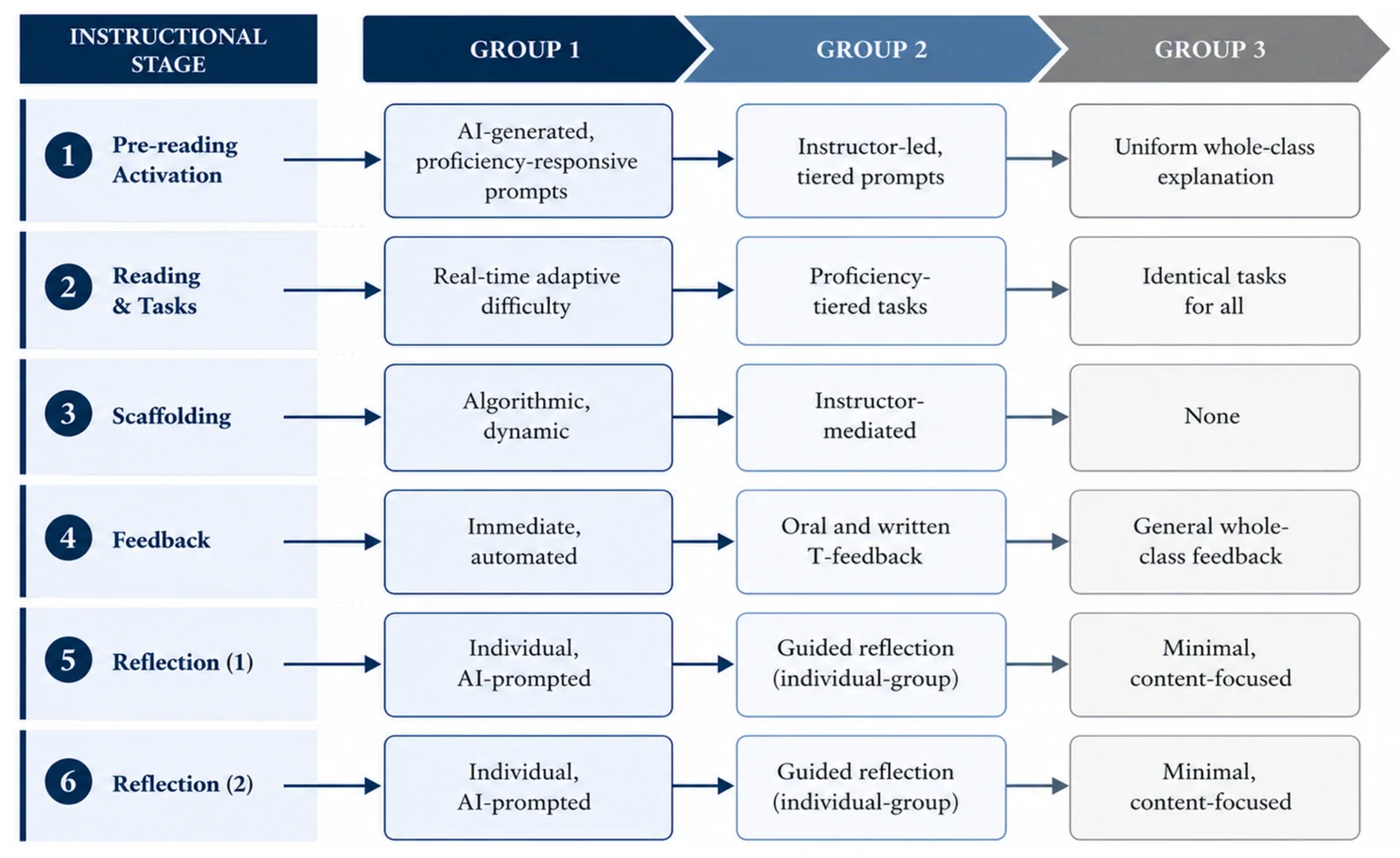
Analytical Comparison of Session-Level Instructional Procedures.

All instructional sessions followed a standardized four-stage structure to control instructional time and time-on-task effects across conditions. Each 60 min session consisted of (a) pre-reading activation (10 min), involving schema activation and topical orientation; (b) guided reading and task engagement (30 min), during which learners completed comprehension-focused reading activities; (c) comprehension consolidation (10 min), focused on reinforcing understanding and clarifying ambiguities; and (d) reflection and feedback (10 min), during which learners reviewed performance and learning strategies. All temporal allocations, instructional stages, and task objectives were held constant across conditions (see Figure 3).

#### 3.6.2. AI-Adaptive Personalized Reading Treatment (G1)

The AI-adaptive condition operationalized personalization as a continuous performance-responsive process. During pre-reading activation, learners received system-generated synthesis tasks and topic prompts calibrated to their proficiency levels. In the guided reading stage, the AI system dynamically adjusted instructional input in real time based on learner performance, including text complexity, lexical support, task difficulty, and feedback specificity. Comprehension tasks progressed from literal to inferential and evaluative levels according to demonstrated performance. During consolidation, learners completed AI-generated synthesis tasks and received immediate automated feedback targeting comprehension accuracy and strategic reading behaviors. In the reflection stage, learners responded to AI-delivered prompts addressing perceived difficulty, instructional comfort, and task clarity.

#### 3.6.3. Teacher-Designed Differentiated Reading Treatment (G2)

The teacher-differentiated condition followed the same session structure while implementing differentiation through instructor-mediated task tiering and scaffolding. Pre-reading activities included proficiency-aligned vocabulary instruction and guided background activation. During guided reading, all learners engaged with the same core texts, but task demands varied by proficiency level: A2 learners completed more literal comprehension tasks, whereas B1 learners engaged in inferential and evaluative activities. The instructor provided contingent scaffolding and individualized support throughout the session. Consolidation activities involved proficiency-appropriate summarization and response tasks, followed by oral and written feedback targeting comprehension and strategy use. Reflection activities focused on task difficulty and reading strategies.

#### 3.6.4. Traditional Non-Differentiated Reading Treatment (G3)

The control condition followed the same session structure but implemented uniform instruction without adaptive or differentiated mechanisms. All learners received identical explanations, reading tasks, scaffolding, and feedback regardless of proficiency level. Sessions consisted of whole-class instruction, identical comprehension activities, and general feedback led by the instructor. Reflection activities were limited to brief content-focused discussions without structured evaluation of learning strategies or instructional experience.

#### 3.6.5. Treatment Fidelity and Contamination Control

**Figure 4:**
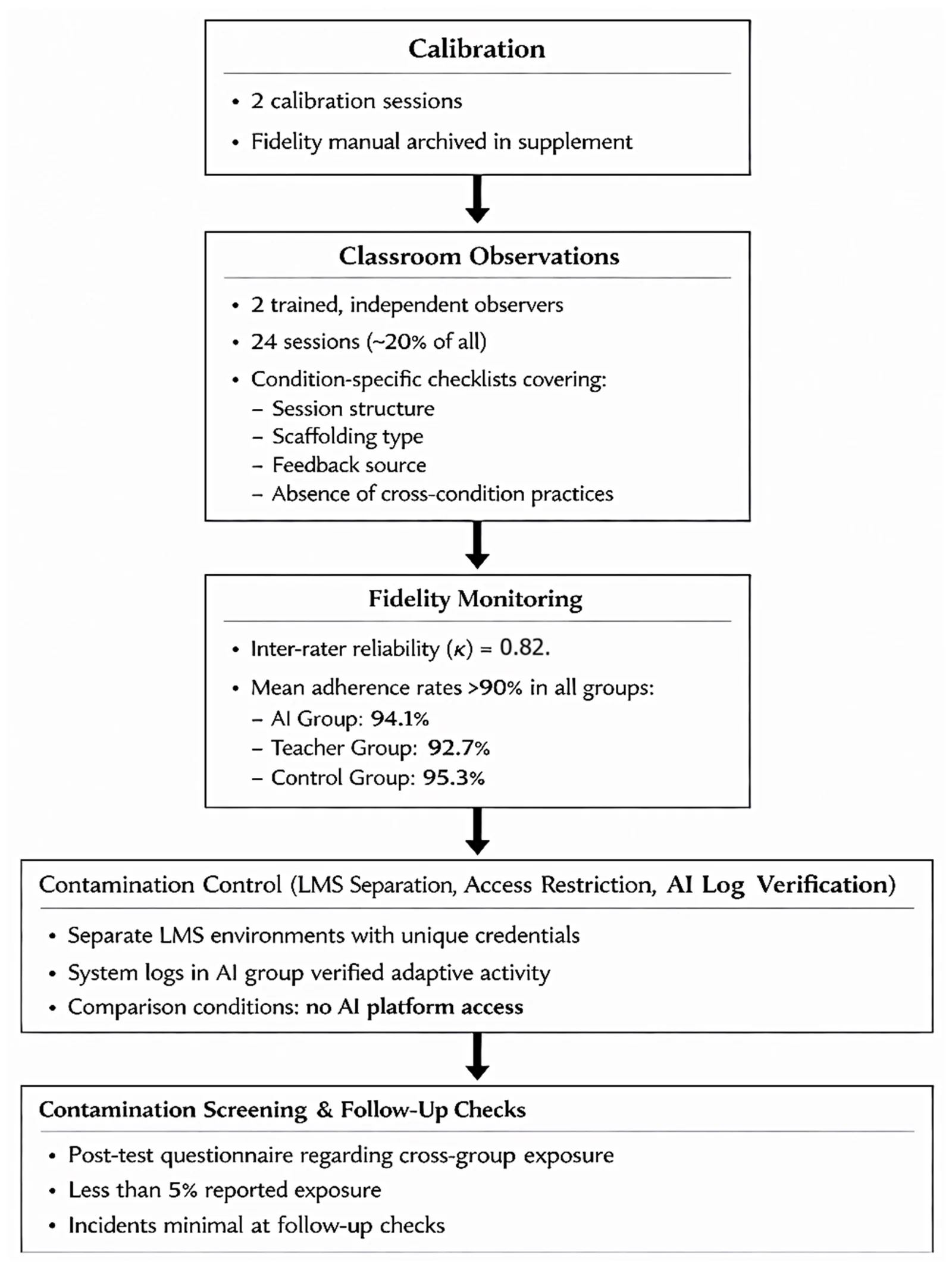
Treatment Fidelity and Contamination Control Procedures Across the 12-Week Intervention.

Treatment fidelity was monitored throughout the 12-week intervention using calibration sessions, structured classroom observations, and a standardized treatment fidelity checklist with condition-specific sections completed independently by two trained observers. The complete fidelity instrument and scoring procedures are provided in the Supplementary Materials. Inter-rater reliability was high (κ = 0.82), and adherence exceeded 90% across all instructional conditions. Contamination was minimized through separate LMS environments, restricted platform access, and AI activity-log verification. Post-test screening indicated minimal cross-condition exposure (<5%). Figure 4 summarizes the fidelity and contamination control procedures.

### 3.7. Reading Comprehension Assessment, Equating, and CEFR Classification

Reading comprehension was assessed using two researcher-constructed parallel-form academic reading tests modeled on the IELTS Academic Reading specification. Because secure operational IELTS materials cannot be reproduced for research dissemination, both forms were developed using publicly available IELTS descriptors, item formats, and construct specifications. Source texts were selected from open-access academic and journalistic materials appropriate for tertiary EFL learners. To enhance content validity, all passages and items were independently reviewed by two experienced EAP instructors and one applied linguistics researcher with IELTS examiner training experience. Reviewers evaluated alignment with the IELTS Academic Reading descriptors, the CEFR reading scales, and the curricular objectives.

Psychometric comparability between forms was established through KR-20 reliability estimation, Rasch modeling, and common-item equating using 12 anchor items. Item functioning, score distributions, and cognitive-domain coverage demonstrated strong comparability across forms, supporting valid measurement of instructional gains. Table 5 summarizes the psychometric and structural characteristics of the two parallel forms.

A detailed assessment blueprint and a parallel-form comparability framework are provided in Appendix B. Because adaptive instructional systems are highly sensitive to measurement quality, the present study treated assessment validity as a foundational design requirement rather than a procedural necessity. Parallel-form equating, Rasch calibration, and CEFR-linked interpretation were implemented to ensure that observed developmental patterns reflected meaningful differences in reading proficiency rather than measurement artifacts. Consequently, this study contributes to ongoing discussions on interpreting assessments in AI-mediated learning environments.

#### 3.7.1. Scoring Procedures

Each test consisted of 40 dichotomously scored items (1 = correct, 0 = incorrect), yielding raw scores ranging from 0 to 40. Raw scores were converted to Rasch-scaled ability estimates to permit interval-level analysis and to support CEFR classification. Two trained raters independently verified the accuracy of the scoring for 20% of randomly selected scripts. Inter-rater agreement exceeded 99%, and all discrepancies were resolved through discussion.

#### 3.7.2. CEFR Mapping and Proficiency Classification

CEFR categories were used as an interpretive framework rather than as independently validated proficiency certifications. Following Rasch calibration and common-item equating, learner ability estimates were placed on a common logit scale. Published IELTS–CEFR correspondence documents (e.g., the British Council, Cambridge Assessment English, and the Council of Europe) informed the development of locally defined Rasch cut-score ranges approximating CEFR proficiency within the present assessment system. These thresholds were used solely for descriptive interpretation and should not be interpreted as equivalent to official CEFR certification or externally validated IELTS scores. Preliminary local validation compared Rasch-based classifications with institutional placement records for a random subsample (*N* = 20), yielding substantial agreement (Cohen’s κ = 0.81). Because institutional placement targets the university’s A2–B1 instructional range, this comparison provides only preliminary support for the local classification framework and does not constitute external validation across the full CEFR continuum (see Table 6).

#### 3.7.3. Interpretive Cautions Regarding Vertical Progression

Although the Rasch-scaled score distribution extended beyond the instructional target range, interpret the resulting B2 and C1 classifications as locally derived Rasch-based categories rather than externally validated CEFR proficiency. Because the assessments were designed primarily to measure development within the institutional A2–B1 range, classifications above this range represent descriptive indicators of relative performance on the local Rasch scale. As no formal CEFR standard-setting study or external CEFR-calibrated assessment was conducted, broader proficiency claims require external validation.

#### 3.7.4. Learner Experience Measures and Psychometric Validation

Learner experience was assessed using an 18-item questionnaire comprising four theoretically defined constructs: Cognitive Load (5 items), Engagement (5 items), Instructional Comfort (4 items), and Perceived Ownership of Reading Achievement (4 items). Responses were recorded on a five-point Likert scale ranging from 1 (Strongly Disagree) to 5 (Strongly Agree). The questionnaire was administered once immediately after the intervention; consequently, it supports comparisons of post-intervention learner perceptions across instructional conditions but not within-participant change over time. The questionnaire was adapted from established educational psychology and computer-assisted language learning measures and reviewed by three experts for content relevance, clarity, and contextual appropriateness for tertiary EFL learners. A test–retest assessment demonstrated satisfactory temporal stability (Spearman’s *r* = 0.878, *p* = 0.001). Internal consistency was acceptable to excellent across all four constructs (Cronbach’s α = 0.81–0.90). The instrument’s internal structure was evaluated using a four-factor confirmatory factor analysis (CFA), with each item loading on its prespecified construct. Because responses were measured on five-point ordinal Likert scales, the model was estimated using the weighted least squares mean- and variance-adjusted (WLSMV) estimator in the *lavaan* package (v0.6-18) in R 4.4.1. Missing data were minimal (<2%), and all participants (N = 87) were retained.

The final four-factor model demonstrated acceptable-to-good fit: χ^2^(129) = 187.42, *p* < 0.001, χ^2^/df = 1.45, CFI = 0.963, TLI = 0.955, RMSEA = 0.071, 90% CI [0.048, 0.092], and SRMR = 0.061. Standardized factor loadings ranged from 0.68 to 0.85 (all *p* < 0.001), providing preliminary support for the intended measurement structure, pending independent replication (see Table 7). Latent factor correlations were moderate and theoretically consistent, with Cognitive Load negatively associated with Engagement (*r* = −0.42), Instructional Comfort (*r* = −0.55), and Perceived Ownership of Reading Achievement (r = −0.31), whereas Engagement was positively associated with Instructional Comfort (r = 0.61) and Perceived Ownership of Reading Achievement (r = 0.58); Instructional Comfort was also positively correlated with Perceived Ownership of Reading Achievement (r = 0.49), supporting satisfactory discriminant validity.

Inspection of modification indices identified one theoretically justified correlated residual between items CL2 and CL4, both reflecting perceived time pressure during reading. Allowing for this residual covariance significantly improved model fit (Δχ^2^ = 12.8, Δdf = 1, *p* < 0.001) without altering the factor structure. No additional modifications were introduced because the remaining indices lacked theoretical justification.

A four-factor confirmatory factor analysis (CFA), conducted on the same sample of 87 participants included in the principal analyses with a modest case-to-parameter ratio, provided preliminary support for the hypothesized measurement structure. The model demonstrated acceptable-to-good fit, χ^2^(129) = 187.42, *p* < 0.001, CFI = 0.963, TLI = 0.955, RMSEA = 0.071 (90% CI [0.048, 0.092]), and SRMR = 0.061. Standardized factor loadings ranged from 0.68 to 0.85, and all loadings were statistically significant (*p* < 0.001), providing preliminary evidence of structural validity that requires independent replication in a larger, independent sample. Detailed CFA results are provided in the Supplementary Materials.

To our knowledge, no previously validated reading-specific measure of AI-mediated perceived ownership of reading achievement was available for tertiary EFL reading contexts. Accordingly, the four-item Perceived Ownership of Reading Achievement subscale was developed through theory-informed adaptation of constructs related to learner agency, ownership, feedback literacy, and academic identity (e.g., Norton, 2013; Carless & Boud, 2018). Item wording was adapted to the context of reading comprehension rather than writing while retaining the theoretical focus on learners’ perceived ownership of their reading processes. Three specialists in applied linguistics and educational measurement independently evaluated the items for conceptual relevance, clarity, and construct alignment. Minor wording revisions were made following expert feedback before pilot testing.

*Conceptual Distinction*

Although conceptually related, perceived ownership of reading achievement differs from several adjacent constructs. Learner autonomy concerns independent regulation of learning decisions; agency refers more broadly to perceived capacity for intentional action; ownership emphasizes psychological investment in learning outcomes; and self-efficacy reflects confidence in one’s capability to perform successfully. By contrast, perceived ownership of reading achievement refers specifically to learners’ perception that successful comprehension remains fundamentally their own intellectual accomplishment despite adaptive instructional support. The construct therefore integrates elements of ownership, agency, and attribution, but is operationalized narrowly as learners’ perceived ownership of their own reading achievement rather than as a broader claim about authorship or identity.

### 3.8. Statistical Analysis Plan

For RQ1, the primary analysis is a descriptive, exploratory comparison of the recoverable pre-to-post trajectories: model-implied pre-test and post-test values for each of the three reported instructional groups (with the two AI-adaptive classrooms analyzed jointly, not as two individually demonstrated trajectories), and model-implied pre-to-post change is reported directly, without treating the classrooms as interchangeable replicates of their assigned instructional condition. Classroom section identifiers (G1a, G1b, G2, G3) were retained throughout the analytic dataset and were used as the clustering factor in the mixed-effects analysis; simple, unmodeled pre-test and post-test descriptive statistics were also computed separately for G1a and G1b. However, instructional condition was entered into the model as a fixed effect shared by both AI-adaptive classrooms, so the reported AI-adaptive coefficients and corresponding model-implied trajectory represent G1a and G1b jointly rather than as two separately estimated classroom effects. Because only two clusters represent the AI-adaptive condition, classroom-specific model-based estimates were not treated as stable inferential estimates or as independent replication of an instructional effect. Accordingly, the primary presentation for RQ1 combines raw classroom-specific descriptives with the pooled model-implied pre-to-post pattern. This presentation does not constitute independent classroom-level replication and cannot provide separable, generalizable estimates of instructional condition effects.

A participant-level 2 × 3 mixed-design ANOVA, with Time (pre-test, post-test) as the within-subject factor and Instructional Condition (AI-adaptive, teacher-differentiated, and non-differentiated) as the between-subject factor, is additionally reported as a secondary, supplementary analysis. Because this analysis treats the 87 learners as independent units despite instructional condition having been assigned at the classroom level, and because it does not model classroom clustering, its Time × Instructional Condition F test, *p*-value, and partial η^2^ should not be treated as primary evidence of an instructional condition effect. The study reports them instead for transparency and comparability with prior participant-level conventions. The exploratory cluster-adjusted mixed-effects model described below serves the same secondary, robustness-checking role and, with only four clusters, cannot overcome the unit-of-analysis limitation.

To assess the robustness of these patterns, an exploratory cluster-adjusted linear mixed-effects model was fitted with repeated observations nested within learners and learners nested within classroom sections. Because only four classroom clusters were available, the model cannot separate instructional effects from cluster membership or provide stable treatment effect estimates. Accordingly, coefficients, confidence intervals, and *p*-values are reported for transparency as sample-specific indicators of direction and uncertainty, not as confirmatory evidence of treatment effectiveness. Statistical significance for the mixed-design ANOVA was evaluated at α = 0.05 (two-tailed), with multiplicity adjustments applied to pairwise comparisons where appropriate. Model assumptions were examined using standardized residuals and Q–Q plots (or Shapiro–Wilk tests) for residual normality and Levene’s test for homogeneity of variance. Because the within-subject factor contained only two levels, sphericity was satisfied automatically, making Mauchly’s test and Greenhouse–Geisser/Huynh–Feldt corrections unnecessary. Effect sizes are reported as partial eta squared (η^2^_p_), with 95% confidence intervals where available.

Preliminary analyses included descriptive statistics, baseline-equivalence testing, outcome-specific intraclass correlation coefficients (ICCs), and calculation of design effects and nominal effective sample sizes. Design effects were calculated using both the conventional formula, DE = 1 + ($\bar{m}$ − 1) ρ, and the unequal-cluster correction, DE = 1 + {[(1 + CV^2^) $\bar{m}$] − 1} ρ. Because classroom sizes were nearly identical (22, 22, 21, and 22 learners; $\bar{m}$ = 21.75; CV ≈ 0.02), both methods produced virtually identical estimates. Effective sample sizes (N/DE) are presented descriptively to illustrate the potential reduction in independent information associated with clustering and should not be interpreted as overcoming the inferential limitations imposed by the four-cluster design. Baseline reading ICCs were estimated from pre-intervention scores, whereas ICCs for cognitive load, engagement, instructional comfort, and perceived ownership of reading achievement were estimated from post-intervention questionnaire data because these outcomes were measured only after the intervention, as shown in Table 8. Appendix C provides full calculation steps for each design effect and effective sample size, including verification using the unequal-cluster-size correction formula.

For RQ2 and the quantitative component of RQ3, learner experience variables were available only at post-test. Because participant-level one-way ANOVAs assume independence and this study included only four classroom clusters (with single clusters representing the teacher-differentiated and non-differentiated conditions), inferential comparisons were not considered sufficiently robust. These outcomes are therefore reported descriptively by instructional condition using means and standard deviations, and are interpreted as classroom-specific patterns rather than causal treatment effects.

The non-differentiated classroom showed an estimated pre-to-post increase of b = 0.89 (*p* < 0.001). Relative to this trajectory, the AI-adaptive classrooms showed an additional estimated increase of b = 0.44 (*p* = 0.002), whereas the teacher-differentiated classroom showed an additional increase of b = 0.29 (*p* = 0.057). The AI classrooms also showed a higher observed baseline score than the control classroom (b = 0.27, *p* = 0.027). Because only four classroom clusters were available, these coefficients describe model-implied baseline contrasts and pre-to-post trajectories and should not be interpreted as separable or generalizable estimates of instructional effectiveness.

Table 9 presents the exploratory cluster-adjusted mixed-effects robustness analysis. Pre-test and the non-differentiated condition served as the reference categories; consequently, the condition main effects represent estimated baseline contrasts, and the Time × Condition terms represent differential pre-to-post trajectories relative to the non-differentiated class. The analysis followed the mixed-design ANOVA to examine whether the observed within-sample trajectory pattern remained evident after accounting for learner and classroom membership. Given the four-cluster design, the coefficients, confidence intervals, and *p*-values are sample-specific indicators of direction and uncertainty and should not be interpreted as separable or generalizable estimates of instructional condition effects.

The class assigned to the non-differentiated condition showed an estimated pre-to-post increase of b = 0.89 (SE = 0.09, *p* < 0.001). Relative to this trajectory, the two classes assigned to the AI-adaptive condition showed an additional estimated increase of b = 0.44 (SE = 0.14, *p* = 0.002), whereas the teacher-differentiated class showed an additional estimated increase of b = 0.29 (SE = 0.15, *p* = 0.057). The AI-versus-control baseline coefficient indicated that the AI-adaptive classes began with a higher estimated reading score than the control class, b = 0.27 (SE = 0.12, *p* = 0.027). These coefficients describe sample-specific contrasts among the model-implied pre-to-post trajectories, distinguishing three instructional groups (with the two AI-adaptive classrooms analyzed jointly) drawn from the four participating clusters, and should not be interpreted as separable or generalizable estimates of instructional condition effects, because instructional condition was partially confounded with classroom membership and the estimates were derived from only four clusters. Sensitivity analyses testing this Time × AI coefficient across alternative model specifications, along with pairwise effect sizes for reading gains, are reported in Appendix D.

## 4. Results

### 4.1. Preliminary Analyses: Baseline Comparability and Initial Imbalance

Before the intervention, continuous reading scores and categorical CEFR distributions were examined across instructional conditions. The CEFR band distributions were broadly similar: the AI-adaptive condition included 23 A2 and 21 B1 learners, the teacher-differentiated condition included 11 A2 and 10 B1 learners, and the non-differentiated control condition included 13 A2 and 9 B1 learners. However, similarity in these categorical distributions did not establish equivalence on the continuous Rasch-scaled reading measure. Because the original participant-level baseline comparison output is no longer available, baseline comparability on the continuous reading measure was evaluated using the available model-based estimates. The exploratory mixed-effects robustness analysis estimated a baseline contrast of b = 0.27 (SE = 0.12, *p* = 0.027) between the AI-adaptive and non-differentiated classes and b = 0.19 (SE = 0.13, *p* = 0.148) between the teacher-differentiated and non-differentiated classes. Given the four-cluster design, these *p*-values are reported for transparency rather than confirmatory inference and should not be used to classify the instructional conditions as definitively equivalent or nonequivalent. Instead, the coefficients indicate that baseline equivalence on the continuous reading measure was not established within this sample.

Baseline equivalence on the continuous reading measure was therefore not established. Accordingly, the primary longitudinal interpretation, consistent with Section 3.8, focuses on the descriptive comparison of the recoverable pre-to-post trajectories, distinguishing three instructional groups (with the two AI-adaptive classrooms analyzed jointly) drawn from the four participating clusters; the Time × Instructional Condition interaction from the mixed-design ANOVA and the baseline-adjusted ANCOVA sensitivity analysis are reported as secondary, supplementary analyses. These evaluate differences in pre-to-post change after accounting for modeled baseline contrasts and are not treated as primary evidence of an instructional condition effect. Consistent with the study design, all of these findings are interpreted as condition-associated patterns within the participating classroom sections rather than definitive treatment effect estimates; this reflects the limited number of classroom clusters and the partial confounding of instructional condition with classroom membership.

### 4.2. RQ1: Recoverable Pre-to-Post Reading Development Patterns Across the Participating Classrooms

#### 4.2.1. Descriptive Presentation of the Recoverable Pre-to-Post Trajectories

Consistent with the classroom-level design, the primary interpretation of RQ1 is descriptive rather than confirmatory. Four classroom sections participated in the study: two AI-adaptive classrooms (G1a and G1b), one teacher-differentiated classroom (G2), and one non-differentiated classroom (G3), as shown in Table 10. Classroom membership was retained throughout the analytic dataset, and simple, unmodeled pre-test and post-test descriptive statistics for G1a and G1b are reported separately in Table 11. However, because only two clusters represent the AI-adaptive condition, a separable, cluster-adjusted model-based trajectory for G1a and G1b individually was not treated as a stable inferential estimate within the mixed-effects framework reported in Table 9, since with only two clusters such an estimate would not be distinguishable from sampling noise; consequently, the model-implied trajectory in Table 10 represents the two AI-adaptive classrooms jointly rather than as two independently modeled classroom trajectories.

For transparent descriptive presentation of the fitted longitudinal pattern, model-implied pre-test, pre-to-post change, and post-test values were reconstructed from the fixed-effect coefficients reported in Table 9. The non-differentiated classroom served as the reference category, with an estimated pre-test value of −0.76 and an estimated pre-to-post change of 0.89. The AI-adaptive baseline contrast was 0.27 and the Time × AI coefficient was 0.44, yielding a model-implied pooled AI-adaptive pre-test value of −0.49, an estimated gain of 1.33, and a post-test value of 0.84. The teacher-differentiated baseline contrast was 0.19 and the Time × Teacher coefficient was 0.29, yielding corresponding values of −0.57 at pre-test, a gain of 1.18, and 0.61 at post-test. Thus, the recoverable descriptive pattern indicates the largest model-implied pre-to-post increase for the two AI-adaptive classrooms analyzed jointly, followed by the teacher-differentiated classroom and the non-differentiated classroom.

Importantly, the values in Table 10 are model-implied values reconstructed from the fitted coefficients rather than raw classroom means; the corresponding raw classroom means for G1a and G1b are reported separately in Table 11. They therefore should not be interpreted as four independently modeled classroom trajectories. Although the descriptive statistics in Table 11 indicate broadly similar pre-test baselines and gain magnitudes for G1a and G1b, the pooled model-based AI-adaptive trajectory in Table 10 cannot formally test, with only two clusters, whether this apparent similarity reflects a genuine absence of classroom-level heterogeneity or is itself an artifact of the limited number of clusters. Moreover, because only four classroom clusters were randomized and the teacher-differentiated and non-differentiated conditions were each represented by a single classroom, instructional condition cannot be separated from classroom-specific influences. Accordingly, these trajectories are presented as sample-specific descriptive patterns and not as separable, confirmatory, or generalizable estimates of instructional effects.

**Figure 5:**
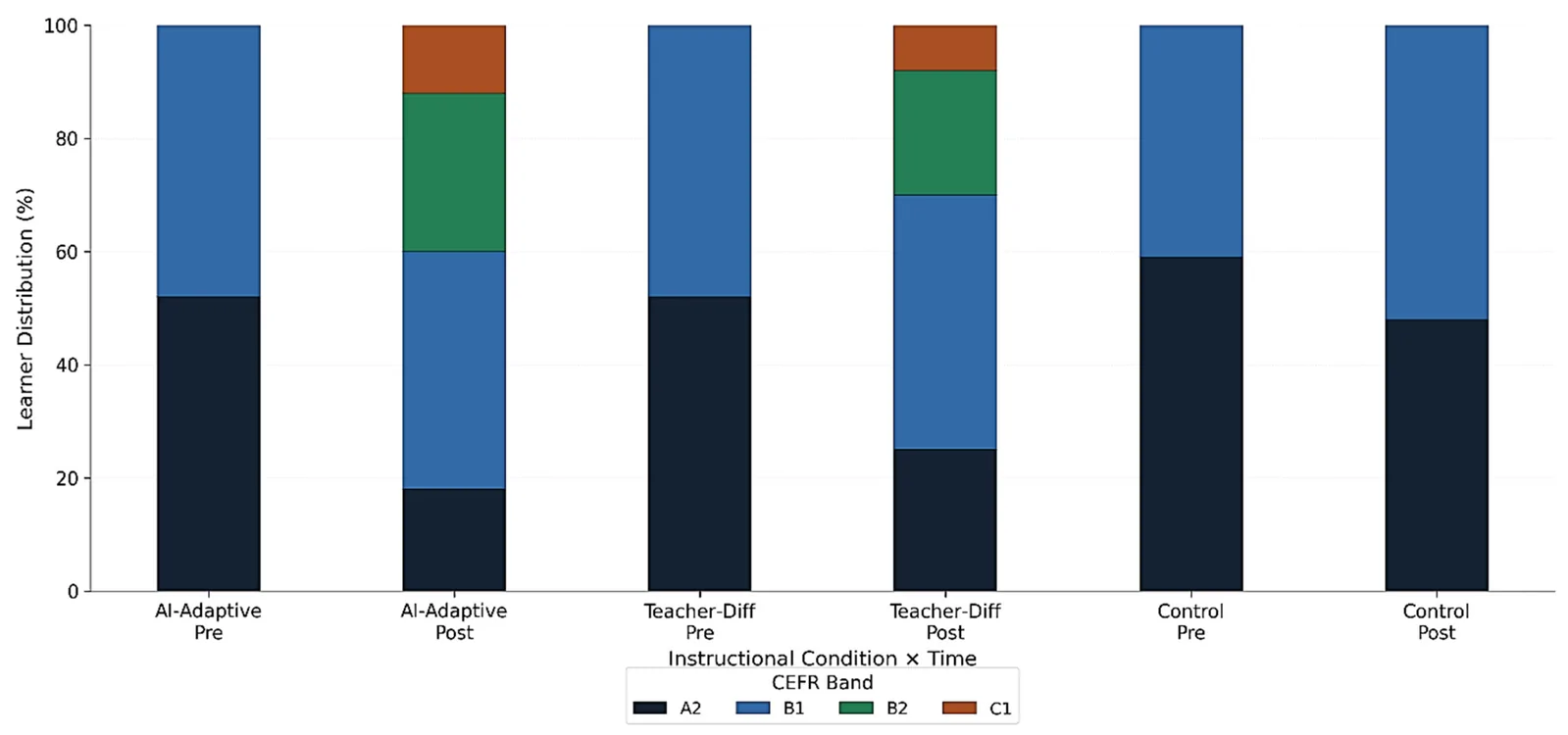
Redistribution of learners across CEFR proficiency bands before and after intervention by instructional condition.

Because the analysis treated learners as independent units and because the teacher-differentiated and non-differentiated conditions were each represented by a single class, the interaction cannot distinguish instructional condition differences from classroom-specific influences. It is therefore interpreted as a secondary, within-sample condition-associated trajectory pattern that supplements, but does not supersede, the primary descriptive comparison of the recoverable model-implied patterns, and it should not be treated as a definitive or generalizable estimate of treatment effects. Post-test results indicated descriptive upward redistribution across CEFR bands within the Rasch-linked classification framework used in the present study. Several learners produced Rasch-scaled score profiles corresponding to higher CEFR classifications than those observed at baseline, although these classifications should be interpreted with caution given the study’s classroom-based design and limited cluster structure (see Figure 5).

**Figure 6:**
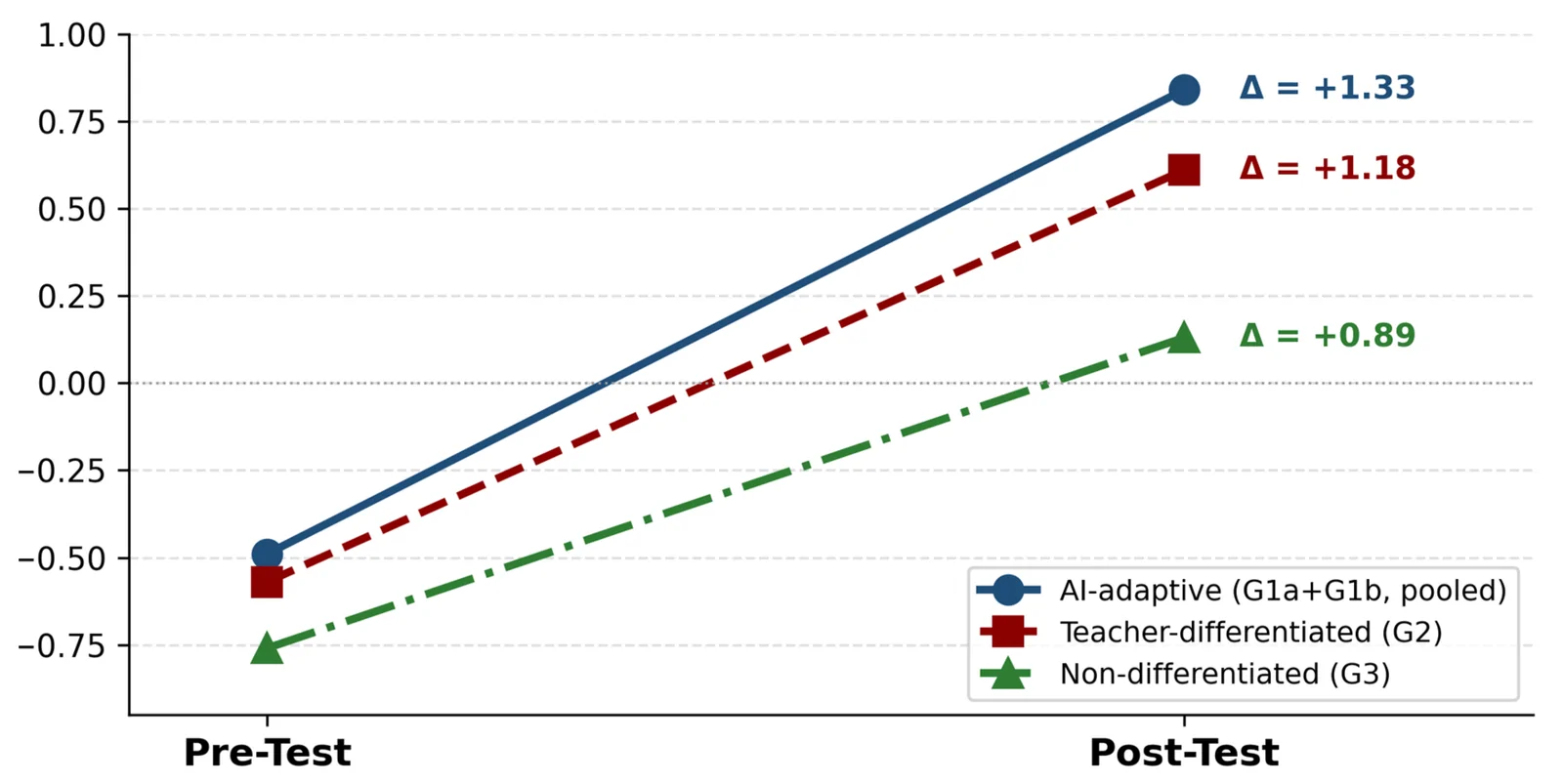
Model-Implied Pre-to-Post Reading Trajectories (Rasch Scale) by Instructional Condition, Consistent with Table 10.

Learners in the class assigned to teacher differentiation also showed observed improvement relative to baseline, with some post-test classifications extending into B2 and C1. Learners in the class assigned to the non-differentiated condition showed a more modest pattern of change, with most classifications remaining within the A2–B1 range and none reaching C1. These descriptive differences characterize only the participating classes. Because each of these conditions was represented by a single class, the patterns cannot be attributed uniquely to teacher differentiation, non-differentiated instruction, maturation, exposure, or other class-specific influences (see Figure 6).

Accordingly, the CEFR redistribution patterns should be interpreted as descriptive indicators of developmental change within the present assessment framework rather than definitive evidence of broader proficiency advancement.

#### 4.2.2. Comparative Effect Magnitude

Effect size estimates described the magnitude and uncertainty of the observed gain-score contrasts among the participating classroom sections. The contrast between learners in the two AI-adaptive classes and learners in the single non-differentiated class was medium-to-large, whereas the contrast between learners in the AI-adaptive classes and learners in the single teacher-differentiated class was small, and its confidence interval crossed zero. Because instructional condition and classroom membership cannot be separated in this four-cluster design, these estimates do not quantify generalizable instructional effects. They are sample-specific descriptive contrasts and should not be used to rank the effectiveness of the three instructional approaches.

### 4.3. RQ2: Learner Experience, Cognitive Load, Engagement, and Instructional Comfort

Post-intervention questionnaire data showed descriptive differences in learner experience across instructional conditions for cognitive load, engagement, instructional comfort, and perceived ownership of reading achievement. Consistent with the Statistical Analysis Plan (Section 3.8), these outcomes were measured only after the intervention, and the study included only four classroom clusters, with single clusters representing the teacher-differentiated and non-differentiated conditions; inferential comparisons across instructional conditions were therefore not considered sufficiently robust and are not reported. Table 12 presents the post-intervention descriptive statistics for learner experience measures using the original 1–5 response scale, reported by instructional condition.

### 4.4. RQ3: Perceived Ownership of Reading Achievement and Ownership of Learning

Qualitative findings complement, rather than verify, the quantitative results by illustrating the range of learner experiences across the three instructional conditions. Although several themes were shared, participants also described differing and occasionally contrasting experiences, reflecting variation in how they perceived the instructional environments. Representative quotations are identified using anonymous participant codes (e.g., AI-12, TD-07, ND-15). Participants in the AI-adaptive classes generally described the adaptive system as supportive rather than controlling, reporting that personalized sequencing made reading appropriately challenging while preserving their sense of interpretive responsibility. Many associated the adaptive support with greater confidence, lower frustration, and stronger perceptions of ownership over their reading performance. As one learner explained, AI-12: *“When the reading became difficult, the system changed slowly, so I didn’t feel suddenly overwhelmed. I still felt that I was the person understanding the text.”* Another commented, *“I liked that the activities matched my level most of the time. It made me feel more confident instead of stressed.”* Several participants also distinguished instructional support from loss of control. As AI-18 reflected: *“The AI gave support, but it didn’t feel like it was doing the work for me. I still had to think carefully about the reading.”* Similarly, another learner stated, *“It felt more like guidance than control. I could still choose how to understand the passage.”*

Learners in the teacher-differentiated class likewise emphasized reassurance, instructional clarity, and interpersonal guidance. Many valued the instructor’s explanations and responsive support during challenging reading tasks. As TD-07 noted: *“Sometimes the teacher’s explanations helped me feel calmer because I could ask questions when I became confused.”* By contrast, participants in the non-differentiated class more frequently described passivity, cognitive overload, and difficulty maintaining engagement. As ND-15 explained: *“Sometimes the reading felt too difficult from the beginning, and I wasn’t sure how to keep up.”* Additional representative quotations are provided in Appendix E. Because both researchers have experience in AI-assisted language learning, they addressed potential interpretive bias by repeatedly comparing themes with the original responses, discussing alternative interpretations, and selecting quotations that represented both convergent and divergent experiences.

Overall, the qualitative findings indicate that many learners in the AI-adaptive classes reported comparatively strong perceptions of interpretive ownership while viewing adaptive support as compatible with learner agency. However, these findings describe the experiences of the four participating classroom sections only and should not be interpreted as evidence that AI-adaptive instruction preserved or enhanced ownership of reading achievement or caused the reported perceptions. Rather, they provide contextual insight into the learner experiences accompanying the quantitative findings and inform the preliminary interpretation of the proposed CAM-AIR framework.

## 5. Discussion

This study examined reading development and learner experience patterns across two classes assigned to AI-adaptive instruction, one class assigned to teacher-differentiated instruction, and one class assigned to non-differentiated instruction. Across the participating classrooms, learners in the AI-adaptive classrooms showed more favorable reading trajectories, lower perceived cognitive load, higher engagement and instructional comfort, and comparatively stronger perceptions of ownership of reading achievement. Collectively, these findings support a preliminary, mechanism-oriented interpretation in which continuous performance-responsive adaptation may be associated with observed gains in learning alongside relatively strong perceptions of learner agency under some instructional conditions. However, because the intervention involved only four intact classroom clusters, instructional conditions could not be separated from class-level influences. Accordingly, the findings should be interpreted as classroom-level patterns rather than definitive treatment effects. Standardized implementation, treatment-fidelity monitoring, baseline measurement, and exploratory cluster-adjusted analysis strengthened the transparency and robustness assessment of the observed patterns, but they did not convert those patterns into separable treatment effect estimates. Replication using substantially larger numbers of randomized classroom clusters is required before causal or generalizable instructional conclusions can be drawn.

### 5.1. AI-Adaptive Personalization and Reading Comprehension Gains (RQ1)

The AI-adaptive classrooms, analyzed jointly, showed a more favorable recoverable pre-to-post reading trajectory than the comparison classrooms, although the sample-specific contrast with the teacher-differentiated classroom was modest, consistent with previous comparisons of adaptive and teacher-mediated personalization (Gevorgyan, 2024; Holmes et al., 2019; Li et al., 2014). Rather than demonstrating superiority over expert teacher differentiation, the findings provide tentative evidence that continuous performance-responsive adaptation may support reading development by maintaining closer alignment between instructional demands and learner capacity. Within this cognitive alignment perspective, adaptive calibration may facilitate comprehension and inferential processing. However, because instructional condition and classroom membership were inseparable, these observations should be interpreted as classroom-level patterns rather than evidence that adaptive instruction itself produced the observed differences.

### 5.2. Cognitive Load, Engagement, and Instructional Comfort (RQ2)

Participants in the AI-adaptive classrooms reported lower perceived cognitive load, along with higher engagement and instructional comfort, than participants in the comparison classrooms. Interpreted through Cognitive Load Theory, these findings are consistent with the possibility that adaptive calibration of instructional demands reduced learners’ overall perceived cognitive effort while supporting engagement (Sweller et al., 2019). Because cognitive load was assessed only as a global perceived construct, however, the findings cannot distinguish among intrinsic, extraneous, and germane load. Likewise, descriptive proficiency differences should be regarded as hypothesis-generating because the study was not designed or statistically powered to evaluate proficiency-by-condition interactions. Contrary to concerns that AI-supported adaptation may promote dependency or fragmented attention, learners generally experienced the adaptive environment as cognitively supportive, whereas experiences in the teacher-differentiated classroom were similarly positive but somewhat more heterogeneous.

### 5.3. Perceived Ownership of Reading Achievement, Agency, and the Personalization–Homogenization Debate (RQ3)

The findings also contribute to ongoing discussions of learner agency in AI-supported education (Bayne, 2015; Selwyn, 2019). Participants in the AI-adaptive classrooms consistently distinguished adaptive support from a loss of interpretive responsibility, describing the system as facilitating rather than replacing their cognitive engagement. Because perceived ownership of reading achievement was measured only after the intervention and no baseline assessment was available, this study cannot determine whether AI-mediated instruction preserved, enhanced, or altered learner agency. Accordingly, these findings describe learner experiences within the participating classrooms rather than causal effects. Nevertheless, they provide empirical context for the personalization–homogenization debate by illustrating how learners experienced AI-mediated personalization in this instructional setting.

### 5.4. Toward a Mechanism-Oriented Understanding of AI in EFL Reading

Taken together, the findings are consistent with, though they do not establish, a preliminary interpretation of AI-adaptive instruction as a mechanism for cognitive alignment rather than as a substitute for pedagogy. Within this perspective, AI automates fine-grained instructional adaptation, whereas pedagogical judgment, curriculum alignment, assessment interpretation, learner motivation, and ethical responsibility remain teacher-mediated. The educational value of AI therefore appears to depend less on automation itself than on the quality of alignment achieved between learner characteristics and instructional support. Accordingly, the proposed Cognitive Alignment Model of AI-Adaptive Reading (CAM-AIR) should be viewed as a preliminary interpretive framework that organizes the observed findings and generates testable hypotheses rather than as a validated explanatory model.

### 5.5. CAM-AIR and Human-in-the-Loop Educational AI

The observed classroom patterns are consistent with a human-in-the-loop interpretation in which AI functioned as a performance-responsive instructional support system within teacher-managed pedagogy rather than as an autonomous instructional agent. Teachers retained responsibility for curriculum alignment, instructional oversight, assessment interpretation, and learning management, while the adaptive system individualized instructional difficulty. These patterns are compatible with the possibility that educational AI can complement rather than substitute for teacher expertise; however, the present four-cluster design does not establish that this arrangement is more effective than alternative instructional models. Future multisite cluster-randomized studies, longitudinal assessment of learner agency, and direct tests of the mechanisms proposed in CAM-AIR are needed to evaluate the framework’s generalizability and explanatory validity.

### 5.6. Limitations and Future Research Directions

Several methodological limitations define this study’s evidential scope. Most importantly, only four intact classroom sections participated, with two allocated to AI-adaptive instruction and only one each to teacher-differentiated and non-differentiated instruction. Consequently, instructional condition could not be disentangled from classroom-specific influences such as peer composition, classroom climate, interactional dynamics, motivation, or localized instructional variation. ICC estimation, design effect calculations, mixed-effects modeling, and sensitivity analyses clarify the potential influence of clustering and the uncertainty surrounding the observed patterns, but they cannot substitute for the independent information provided by a substantially larger number of randomized classroom clusters or produce definitive treatment effect estimates.

A related limitation concerns the inferential, rather than descriptive, separability of the two AI-adaptive classrooms. Classroom membership (G1a, G1b) was retained throughout the analytic dataset, and simple pre-test and post-test descriptive summaries for each classroom are reported separately in Table 11; these raw means indicate broadly similar baseline scores and gain magnitudes for G1a and G1b (pre-test M = −0.44, SD = 0.53 for G1a and M = −0.54, SD = 0.38 for G1b; post-test M = 0.93, SD = 0.82 for G1a and M = 0.75, SD = 0.72 for G1b; mean gain M = 1.37, SD = 0.71 for G1a and M = 1.29, SD = 0.71 for G1b). However, because only two clusters represent the AI-adaptive condition, a statistically separable, cluster-adjusted trajectory for each classroom individually was not treated as a stable inferential estimate within the mixed-effects framework, and the reading development pattern was therefore represented jointly in the model-implied trajectory reported in Table 10. This limits formal assessment of whether the two AI-adaptive classrooms are statistically distinguishable in their patterns of change and further limits claims of replication at the classroom level. The learner experience questionnaire also requires cautious interpretation. Its four-factor structure was examined using the same N = 87 sample included in the principal analyses, and the case-to-parameter ratio for the 18-item model was modest. The psychometric findings therefore provide preliminary evidence of structural validity rather than independent validation and should be replicated in larger external samples. In addition, the learner experience measures were self-reported and may have been influenced by perceptual bias, novelty effects, or social desirability. The qualitative component likewise represents learners’ reported experiences rather than independent evidence of the mechanisms proposed in CAM-AIR.

This study was conducted at a single institution with first-year female university EFL learners, limiting generalizability across genders, educational levels, linguistic contexts, and instructional cultures. Although the intervention extended over 12 weeks and used parallel-form Rasch-equated assessments, no delayed follow-up was conducted, leaving the durability and transfer of the observed reading development patterns uncertain. Furthermore, the locally derived Rasch-linked CEFR categories were not established through independent CEFR standard setting or external scale linking; classifications beyond the institutional A2-B1 instructional range should therefore be interpreted descriptively rather than as externally validated proficiency levels.

Finally, neither homogenization nor the proposed cognitive-alignment mechanisms were directly operationalized. The comparison also involved instructional architectures that differed simultaneously in adaptation source, responsiveness, continuity, feedback density, and personalization granularity. The present design therefore cannot identify which component, if any, accounts for the observed classroom patterns. Future research should employ multisite cluster-randomized designs with substantially more, more evenly allocated classroom clusters; independent psychometric validation of learner experience measures; repeated and delayed assessment; and more diverse learner populations. Factorial or component-based designs would be particularly useful for separating the contributions of adaptation source, frequency, feedback density, and personalization granularity, while direct measurement of cognitive alignment, learner agency, and learning pathway convergence would provide stronger tests of CAM-AIR and the personalization–homogenization hypothesis.

## 6. Conclusions

This study compared AI-adaptive instruction, teacher-differentiated instruction, and non-differentiated instruction in relation to EFL learners’ reading development and learning experiences. Across the participating classroom sections, learners in the AI-adaptive classrooms showed more favorable reading trajectories, lower perceived cognitive load, higher engagement and instructional comfort, and comparatively stronger perceptions of ownership of reading achievement. The teacher-differentiated classroom also showed meaningful observed improvement, a pattern consistent with the continuing pedagogical value of human-mediated adaptive instruction. Because this study involved only four randomized classroom clusters within a single institutional context, these findings should be interpreted as classroom-level patterns rather than definitive estimates of treatment effectiveness.

Within this sample, learners in the AI-adaptive classrooms showed broader observed redistribution across the locally defined Rasch-linked CEFR categories, including movement into higher categories not observed at baseline; because instructional condition and classroom membership were confounded, this pattern describes the participating classes rather than a demonstrated effect of the instructional approach itself. However, any equity-related interpretation, particularly regarding lower-proficiency learners, remains exploratory because this study was not designed or statistically powered to evaluate proficiency-by-condition interactions. Likewise, qualitative findings indicated that learners generally experienced adaptive support as facilitating rather than replacing their own interpretive effort and responsibility. Although these observations provide empirical context for the personalization–homogenization debate, homogenization itself was not directly operationalized or behaviorally measured. The findings also provide preliminary interpretive support for the Cognitive Alignment Model of AI-Adaptive Reading (CAM-AIR) as a framework for understanding the relationships among adaptive instructional support, perceived cognitive load, learner experience, and agency in AI-mediated reading environments. CAM-AIR should, however, be regarded as a hypothesis-generating interpretive framework rather than a validated explanatory model.

Overall, the classroom-level patterns are consistent with the possibility that transparent, performance-responsive AI may support reading development and learner engagement when implemented within carefully designed, teacher-managed instructional environments.

## Figures and Tables

**Table 1:** Participant Demographic and Educational Characteristics.

Variable	Description
Total participants	87
Gender	Female
Age range	18–21 years
Educational level	First-year university
Instructional context	English Language Center
Prior English exposure	6–8 years
Target proficiency	CEFR A2–B1 (reading)

**Table 2:** Participant Distribution and Baseline CEFR Band Composition Across Instructional Conditions.

Condition	*N*	B1 (=1)	*p* (Mean)	Population SD	Sample SD
AI-adaptive personalized reading	44	21	0.4773	0.4995 → 0.500	0.5053 → 0.505
Teacher-differentiated reading	21	10	0.4762	0.4994 → 0.500	0.5118 → 0.512
Non-differentiated control	22	9	0.4091	0.4917 → 0.492	0.5032 → 0.503
Total	87	40	0.4598	0.4984 → 0.498	0.5013 → 0.501

**Table 3:** Comparative Overview of Instructional Conditions.

Dimension	AI-Adaptive	Teacher-Differentiated	Non-Differentiated
Source passages	Common source passages	Common source passages	Common source passages
Linguistic realization	Adaptively modified	Teacher-adapted	Unmodified
Learning objectives	Same	Same	Same

**Table 4:** Instructional Treatment Parameters.

Parameter	Specification
Total duration	12 weeks
Treatment phase	Weeks 2–11
Sessions per week	3
Session length	60 min
Total instructional hours	30 per group
Instructor	Same across conditions
Variable manipulated	Mode of adaptation

**Table 5:** Psychometric and Structural Comparability of Parallel Reading Forms.

Indicator	Pre-Test Form	Post-Test Form
Total items	40	40
Number of passages	3	3
Mean passage length	≈890 words	≈905 words
Lexical coverage target	CEFR A2–B2	CEFR A2–B2
KR-20 reliability	0.86	0.88
Rasch person reliability	0.83	0.85
Rasch item reliability	0.94	0.95
Mean Rasch item difficulty (logits)	0.01	−0.03
SD of item difficulty (logits)	0.88	0.91
Item difficulty range (logits)	−1.72 to +1.81	−1.69 to +1.84
Separation index (persons/items)	1.95/2.65	2.01/2.70
Misfitting items (infit/outfit > 1.5 MNSQ)	0	0
Anchor items (common items for equating)	12	12
Mean anchor difficulty difference	—	<0.20 logits
Literal retrieval items	14	13
Local inference items	8	9
Global inference items	7	7
Vocabulary-in-context items	6	6

**Table 6:** CEFR Classification Bands Based on Rasch-Scaled Score Ranges.

CEFR Level	Rasch-Scaled Score Range
A2	≤−0.80
B1	−0.79 to 0.30
B2	0.31 to 1.10
C1	≥1.11

**Table 7:** Psychometric Properties of Learner Experience Questionnaire.

Construct	Items	Standardized Loading Range	Cronbach’s α
Cognitive Load	5	0.69–0.82	0.88
Engagement	5	0.68–0.85	0.86
Instructional Comfort	4	0.70–0.81	0.81
Perceived Ownership of Reading Achievement	4	0.72–0.83	0.90

**Table 8:** Intraclass Correlation Coefficients (ICCs) and Design Adjustments for Baseline Reading Outcomes and Post-Intervention Learner Experience Measures.

Outcome Variable	Measurement Occasion	ICC	Mean Cluster Size	Conventional DE	Unequal-Size DE	Nominal Effective N
Reading score	Pre-intervention	0.07	21.75	2.453	2.453	35.5
Cognitive load	Post-intervention	0.05		2.038	2.038	42.7
Engagement		0.04		1.830	1.830	47.5
Instructional comfort		0.04		1.830	1.830	47.5
Perceived ownership of reading achievement		0.03		1.623	1.623	53.6

**Table 9:** Exploratory Cluster-Adjusted Mixed-Effects Estimates of Baseline Contrasts and Differential Pre-to-Post Trajectories (Secondary, Robustness Analysis).

Fixed Effect	b	SE	t	*p*	Interpretation
Intercept: Control at pre-test	−0.76	0.10	−7.60	<0.001	Estimated control pre-test mean
AI vs. control at pre-test	0.27	0.12	2.25	0.027	Baseline condition difference
Teacher vs. control at pre-test	0.19	0.13	1.46	0.148	Baseline condition difference
Time: control pre-to-post change	0.89	0.09	9.61	<0.001	Change in control condition
Time × AI	0.44	0.14	3.14	0.002	Additional AI change relative to control
Time × teacher	0.29	0.15	1.93	0.057	Additional teacher group change relative to control

***Note.*** The model is reported as an exploratory robustness analysis. With only four classroom clusters and only one cluster in each of the teacher-differentiated and non-differentiated conditions, the coefficients, confidence intervals, and *p*-values cannot provide stable, separable, or generalizable estimates of instructional condition effects. They describe the direction and uncertainty of the trajectories observed in the participating classes.

**Table 10:** Model-Implied Pre-to-Post Reading Trajectories for the Three Reported Instructional Groups (G1a and G1b Analyzed Jointly).

Participating Classroom (s)	Instructional Condition	Model-Implied Pre-Test	Model-Implied Pre-to-Post Change	Model-Implied Post-Test
G1a + G1b (pooled)	AI-adaptive personalized reading	−0.49	+1.33	0.84
G2	Teacher-differentiated reading	−0.57	+1.18	0.61
G3	Non-differentiated control	−0.76	+0.89	0.13

**Table 11:** Simple (Unmodeled) Pre-Test and Post-Test Descriptive Statistics for the Two AI-Adaptive Classrooms (G1a, G1b).

Classroom	N	Pre-Test M (SD)	Post-Test M (SD)	Mean Gain M (SD)
G1a	22	−0.44 (0.53)	0.93 (0.82)	1.37 (0.71)
G1b	22	−0.54 (0.38)	0.75 (0.72)	1.29 (0.71)

***Note.*** Values are simple, unmodeled classroom means computed directly from the raw pre-test and post-test dataset (not derived from the mixed-effects model reported in Table 9). Gain was computed per learner as post-test minus pre-test score, then averaged within classroom.

**Table 12:** Descriptive Post-Intervention Learner Experience Scores by Instructional Group (Two AI-Adaptive Classrooms Analyzed Jointly).

Measure (1–5 Scale)	AI-Adaptive Personalized Reading: M ± SD	Teacher-Differentiated Reading: M ± SD	Non-Differentiated Control: M ± SD
Cognitive Load	2.01 ± 0.54	2.47 ± 0.61	3.18 ± 0.66
Engagement	4.32 ± 0.48	3.91 ± 0.52	3.08 ± 0.59
Instructional Comfort	4.28 ± 0.50	3.76 ± 0.58	3.02 ± 0.63
Perceived Ownership of Reading Achievement	4.62 ± 0.31	2.03 ± 0.49	3.76 ± 0.91

## Data Availability

The data presented in this study are available on request from the corresponding author. The data are not publicly available due to ethical considerations and the need to protect participants’ privacy and confidentiality.

## Supplementary Materials

The following supporting information can be downloaded at: https://www.mdpi.com/article/10.3390/bs16091478/s1: Supplementary File S0: Ethics Approval Documentation; Table S2.1: Fidelity Checklist Instrument; Table S3.1: Architecture and Functional Modules; Table S3.2: Learner-State Indicators and Operational Definitions; Table S3.3: Adaptable Instructional Dimensions; Table S3.4: Threshold Rules for Real-Time Adaptation; Table S3.5: Parameter Ranges and Stability Constraints; Table S4.1 (Table C1): Parallel-Form Assessment Blueprint and Comparability Framework; Section S5: The 18-Item Learner-Experience Questionnaire: Full Item Pool, Administration, and Scoring; Table S6.1: Overall Confirmatory Factor Analysis Model Fit; Table S6.2: Standardized Factor Loadings; Table S6.3: Latent Factor Correlations; Table S7.1: Sensitivity Analyses Across Alternative Specifications; Table S7.2: Pairwise Effect Sizes for Reading Gains; Table S8.1: Thematic Patterns in Learner Reflections Across Instructional Conditions.

## Appendix A. Multilayered Specification of the AI-Adaptive Reading System

**Table A1:** System Architecture and Functional Modules.

Layer	Module	Primary Function	Inputs	Outputs
L1	Learner Analytics Engine	Computes learner-state metrics in real time	Responses, timing, navigation logs	Accuracy, latency, error indices
L2	Cognitive Alignment Module	Estimates learner–task fit	Metrics from L1	Alignment score
L3	Rule-Based Adaptation Engine	Applies deterministic thresholds	Alignment + CEFR tier	Adaptation commands
L4	Instructional Generator	Modifies texts, tasks, and scaffolds	Adaptation commands	Personalized materials
L5	Logging & Audit Layer	Stores all system decisions	All layers	Replication files

**Table A2:** Learner-State Indicators and Operational Definitions.

Indicator	Computation	Rolling Window	Cognitive Interpretation
Accuracy Rate	Correct/total responses	5 items	Mastery vs. overload
Response Latency	Mean seconds per item	5 items	Processing demand
Error Pattern Index	Lexical/inferential/discourse errors	Session	Skill deficit
Help Requests	Hint activations	Session	Dependency
Backtracking Events	Navigation reversals	Session	Monitoring effort
Self-Reported Difficulty	1–5 scale	Session	Subjective load

**Table A3:** Adaptable Instructional Dimensions.

Dimension	Adjustable Features	Purpose
Text Complexity	Lexical bands, clause depth, cohesion markers	Control intrinsic load
Lexical Support	Glosses, L1 hints, examples	Reduce decoding burden
Scaffolding Intensity	Prompts, outlines, and previews	Support comprehension
Task Difficulty	Literal → inferential → evaluative	Promote depth
Feedback Specificity	Binary → explanatory → strategic	Build metacognition

**Table A4:** Primary Threshold Rules for Real-Time Adaptation.

Trigger Condition	Threshold	Action	Cognitive Rationale
Sustained high accuracy	≥0.80 for 8 items	Increase tier	Under-challenge
Sustained low accuracy	≤0.60 for 5 items	Decrease tier	Overload
Slow responses	+1 SD from baseline	Add scaffolding	Processing strain
Frequent lexical errors	>40% lexical	Increase glossing	Vocabulary gap
Inferential errors	>40% inferential	Reduce cohesion demands	Discourse overload
Low help + high accuracy	<10% + ≥0.80	Fade scaffolds	Independence
High help	>30%	Maintain tier	Avoid oscillation

**Table A5:** Parameter Ranges and Stability Constraints.

Parameter	Lower Bound	Upper Bound	Constraint
Lexical frequency	2000 families	8000 families	CEFR-aligned
Sentence length	12 words	28 words	Syntax ceiling
Inferential prompts	1/paragraph	4/paragraph	Avoid overload
Gloss density	1/40 words	1/10 words	Fade gradually
CEFR shift	−1 band	+1 band	No jumps
Session change	0.5 SD	1 SD	Anti-oscillation

## Appendix B. Parallel-Form Assessment Blueprint and Comparability Framework

**Table A6:** Parallel-Form Assessment Blueprint and Comparability Framework.

Component	Pre-Test Form	Post-Test Form	Comparability Control
Number of passages	3	3	Matched structure
Total items	40	40	Equivalent item load
Mean passage length	≈890 words	≈905 words	Comparable text length
CEFR target range	A2–B2	A2–B2	Same proficiency coverage
Primary genres	Informational/academic	Informational/academic	Genre-matched
Lexical profile	CEFR-aligned academic vocabulary	CEFR-aligned academic vocabulary	Comparable lexical coverage
Syntactic complexity	Moderate clause density and mixed sentence structures	Moderate clause density and mixed sentence structures	Comparable syntactic complexity
Literal retrieval items	14	13	Balanced domain coverage
Local inference items	8	9	Comparable inferential demand
Global inference items	7	7	Comparable higher-order inference demands
Vocabulary-in-context items	6	6	Equivalent lexical reasoning demands
Anchor items for equating	12	12	Common-item Rasch equating
Item difficulty range	−1.72 to +1.81 logits	−1.69 to +1.84 logits	Difficulty alignment
Expert review	2 EAP specialists + 1 IELTS-trained applied linguist	Same	Content validation consistency
Sample task types	Multiple choice, matching, inference, vocabulary interpretation	Same	Format equivalence

## Appendix C. Design Effect, Effective Sample Size, and Cluster Design Verification

**Table A7:** Design Effect and Effective Sample Size Calculations for Each Outcome Measure.

Outcome	ICC	Design Effect Calculation	DE	Approximate Effective Sample Size
Reading score	0.07	DE = 1 + (21.75 − 1)(0.07) = 2.453	2.453	N_eff = 87/2.453 = 35.5
Cognitive load	0.05	DE = 1 + (21.75 − 1)(0.05) = 2.038	2.038	N_eff = 87/2.038 = 42.7
Engagement	0.04	DE = 1 + (21.75 − 1)(0.04) = 1.830	1.830	N_eff = 87/1.830 = 47.5
Instructional comfort	0.04	DE = 1 + (21.75 − 1)(0.04) = 1.830	1.830	N_eff = 87/1.830 = 47.5
Perceived ownership of reading achievement	0.03	DE = 1 + (21.75 − 1)(0.03) = 1.623	1.623	N_eff = 87/1.623 = 53.6

**Table A8:** Verification of the Unequal-Cluster Design Effect.

Parameter	Value
Cluster sizes	22, 22, 21, 22
Mean cluster size ($\bar{m}$)	21.75
Population SD of cluster size	≈0.433
Coefficient of variation (CV)	CV = 0.433/21.75 ≈ 0.0199
Reading ICC	0.07
Unequal-cluster design effect formula	DE = 1 + [(CV^2^ + 1) $\bar{m}$ − 1] × ICC
Calculated unequal-cluster DE	2.4531
Conventional DE	2.4525
Absolute difference	0.0006

## Appendix D. Sensitivity Analyses and Pairwise Effect Sizes for Reading Gains

**Table A9:** Sensitivity Analyses Across Alternative Specifications.

Model Specification	Time × AI Estimate	*p*
Mixed-effects (secondary)	0.44	0.002
Without a class random effect	0.46	0.001
Raw scores	0.41	0.003
ANCOVA	0.39	0.005

**Table A10:** Pairwise Effect Sizes for Reading Gains.

Comparison	Cohen’s d	95% CI	Magnitude
AI vs. Control	0.69	0.16, 1.22	Medium–large
Teacher vs. Control	0.52	0.01, 1.03	Medium
AI vs. Teacher	0.22	−0.30, 0.73	Small

## Appendix E. Thematic Patterns and Representative Learner Reflections

**Table A11:** Thematic Patterns in Learner Reflections Across Instructional Conditions.

Theme	Representative Quotation
Autonomy Through Adaptive Clarity	“The system adjusted when I struggled, but I still felt the answers came from my understanding.” (AI-Adaptive learner)
Reduced Cognitive Pressure	“The reading became easier step by step, so I didn’t panic when texts became difficult.” (AI-Adaptive learner)
Guided but Teacher-Dependent Confidence	“The teacher explained things clearly, but I still waited for help before moving on.” (Teacher-Differentiated learner)
Passive Task Compliance	“Sometimes the reading felt too difficult, and I just followed the class without really understanding.” (Control learner)
Preserved Ownership of Achievement	“Even with support, I felt the improvement was mine because I was still doing the reading myself.” (AI-Adaptive learner)
Predictability as Psychological Safety	“I liked that the tasks matched my level instead of suddenly becoming confusing.” (AI-Adaptive learner)
