# Supplementary material for: Personalization and the Homogenization Debate in AI-Adaptive EFL Reading: Cognitive Load, Learner Agency, and Reading Development
Source: Behav Sci (Basel). 2026 Aug 25;16(9):1478. doi: 10.3390/bs16091478 (PMC13603526; doi:10.3390/bs16091478)
Supplement: Supplementary file 1 [file behavsci-16-01478-s001.zip › behavsci-4396627-supplementary.pdf]

Supplementary Materials

This supplementary file consolidates all appendices and supporting materials referenced in the main manuscript, including the treatment fidelity and contamination-control procedures (Section 3.6.5, Figure 3), the technical architecture and adaptive decision rules of the AI-Adaptive Reading System (Appendix A), the parallel-form assessment blueprint (Appendix C), exploratory sensitivity and robustness analyses (Appendix B), and representative qualitative quotations (Appendix D). The ethics approval letter previously supplied to reviewers should be retained as Supplementary File S0.

S1. Ethics Approval Documentation: The Institutional Review Board / Ethics Committee approval letter for this study is provided as a separate file (previously submitted to reviewers). It is referenced here for completeness and should be included alongside this document as Supplementary File S0 in the final supplementary package.

S2. Treatment Fidelity and Contamination-Control Procedures: As described in Section 3.6.5 of the main text, treatment fidelity was monitored throughout the 12-week intervention using calibration sessions, structured classroom observations, and condition-specific fidelity checklists administered by two independent observers. Inter-rater reliability was high ($\kappa = .82$), and adherence exceeded 90% across all instructional conditions. Contamination was minimized through separate LMS environments, restricted platform access, and AI activity-log verification. Post-test screening indicated minimal cross-condition exposure (<5%). Figure 2 summarizes the fidelity and contamination-control procedures across the intervention period.

Table S2.1. Fidelity Checklist Instrument

Purpose and Instructions

This checklist operationalizes treatment fidelity monitoring across the three instructional conditions of the 12-week reading intervention: (G1) AI-Adaptive Personalized Reading, (G2) Teacher-Designed Differentiated Reading, and (G3) Traditional Non-Differentiated Reading. It is designed to be completed by trained, independent classroom observers following calibration sessions. Each 60-minute session is observed across four standardized stages: pre-reading activation (10 min), guided reading and task engagement (30 min), comprehension consolidation (10 min), and reflection and feedback (10 min). Observers should first complete the Core Session Structure section (common to all conditions), then complete only the condition-specific section that matches the observed class, followed by the Contamination-Control section and the Adherence Scoring Summary. Rate each indicator as Fully Observed,

Partially Observed, Not Observed, or Not Applicable (N/A). Two independent observers should complete this checklist for the same session periodically throughout the intervention to permit inter-rater reliability estimation (e.g., Cohen's kappa).

Session Information

**Instructional Condition**	☐ G1a ☐ G1b (AI-Adaptive) ☐ G2 (Teacher-Diff.) ☐ G3 (Control)	**Session / Week #**	
**Date**		**Class / Section Code**	
**Observer Name / ID**		**Session Start / End Time**	

Section A. Core Session Structure (All Conditions)

#	Fidelity Indicator	Fully Observed	Partially Observed	Not Observed	N/A	Observer Notes
A1	Session began with pre-reading activation (schema activation / topical orientation) and lasted approximately 10 minutes	☐	☐	☐	☐	
A2	Guided reading and task engagement stage lasted approximately 30 minutes and involved comprehension-focused reading activities	☐	☐	☐	☐	
A3	Comprehension consolidation stage lasted approximately 10 minutes and reinforced understanding / clarified ambiguities	☐	☐	☐	☐	
A4	Reflection and feedback stage lasted approximately 10 minutes and involved review of performance and learning strategies	☐	☐	☐	☐	
A5	Total session time and stage sequencing matched the standardized protocol (no stage skipped, reordered, or substantially shortened/extended)	☐	☐	☐	☐	
A6	Task objectives for the session matched those specified in the instructional protocol for this week	☐	☐	☐	☐	

Section B. Condition-Specific Indicators — G1: AI-Adaptive Personalized Reading

Pre-Reading Activation

#	Fidelity Indicator	Fully Observed	Partially Observed	Not Observed	N/A	Observer Notes
B1	Learners received AI-delivered vocabulary and topic prompts calibrated to their individual proficiency levels	☐	☐	☐	☐	

Guided Reading and Task Engagement

#	Fidelity Indicator	Fully Observed	Partially Observed	Not Observed	N/A	Observer Notes
B2	AI system dynamically adjusted instructional input in real time based on learner performance	☐	☐	☐	☐	
B3	Adjustments were evident across text complexity, lexical support, task difficulty, and/or feedback specificity	☐	☐	☐	☐	
B4	Comprehension tasks progressed from literal to inferential to evaluative levels according to demonstrated performance	☐	☐	☐	☐	

Comprehension Consolidation

#	Fidelity Indicator	Fully Observed	Partially Observed	Not Observed	N/A	Observer Notes
B5	Learners completed AI-delivered synthesis tasks	☐	☐	☐	☐	
B6	Learners received immediate automated feedback targeting comprehension accuracy and strategic reading behaviors	☐	☐	☐	☐	

Reflection and Feedback

#	Fidelity Indicator	Fully Observed	Partially Observed	Not Observed	N/A	Observer Notes
B7	Learners responded to AI-delivered prompts addressing perceived difficulty, instructional comfort, and task clarity	☐	☐	☐	☐	

#	Fidelity Indicator	Fully Observed	Partially Observed	Not Observed	N/A	Observer Notes
B8	No teacher-mediated tiering or manual scaffolding was substituted for the AI-adaptive mechanism	☐	☐	☐	☐	

Section C. Condition-Specific Indicators — G2: Teacher-Designed Differentiated Reading

Pre-Reading Activation

#	Fidelity Indicator	Fully Observed	Partially Observed	Not Observed	N/A	Observer Notes
C1	Learners received proficiency-aligned vocabulary instruction and guided background activation	☐	☐	☐	☐	

Guided Reading and Task Engagement

#	Fidelity Indicator	Fully Observed	Partially Observed	Not Observed	N/A	Observer Notes
C2	All learners engaged with the same core texts	☐	☐	☐	☐	
C3	Task demands varied by proficiency level (e.g., A2 learners completed more literal tasks; B1 learners engaged in inferential/evaluative tasks)	☐	☐	☐	☐	
C4	Instructor provided contingent scaffolding and individualized support throughout the session	☐	☐	☐	☐	

Comprehension Consolidation

#	Fidelity Indicator	Fully Observed	Partially Observed	Not Observed	N/A	Observer Notes
C5	Consolidation activities involved proficiency-appropriate summarization and response tasks	☐	☐	☐	☐	
C6	Learners received oral and/or written feedback targeting comprehension and strategy use	☐	☐	☐	☐	

Reflection and Feedback

#	Fidelity Indicator	Fully Observed	Partially Observed	Not Observed	N/A	Observer Notes
C7	Reflection activities focused on task difficulty and reading strategies	☐	☐	☐	☐	
C8	No AI-adaptive tools were used in place of instructor-mediated differentiation	☐	☐	☐	☐	

Section D. Condition-Specific Indicators — G3: Traditional Non-Differentiated Reading

Pre-Reading Activation

#	Fidelity Indicator	Fully Observed	Partially Observed	Not Observed	N/A	Observer Notes
D1	All learners received identical explanations and orientation activities regardless of proficiency level	☐	☐	☐	☐	

Guided Reading and Task Engagement

#	Fidelity Indicator	Fully Observed	Partially Observed	Not Observed	N/A	Observer Notes
D2	Whole-class instruction was used with identical comprehension activities for all learners	☐	☐	☐	☐	
D3	No adaptive or differentiated mechanisms (AI-based or instructor-tiered) were introduced	☐	☐	☐	☐	

Comprehension Consolidation

#	Fidelity Indicator	Fully Observed	Partially Observed	Not Observed	N/A	Observer Notes
D4	General (non-individualized) feedback was provided, led by the instructor	☐	☐	☐	☐	

Reflection and Feedback

#	Fidelity Indicator	Fully Observed	Partially Observed	Not Observed	N/A	Observer Notes
D5	Reflection was limited to brief, content-focused discussion without structured evaluation of learning strategies or instructional experience	☐	☐	☐	☐	
D6	No differentiation, tiering, or AI-adaptive elements were introduced at any stage	☐	☐	☐	☐	

Section E. Contamination-Control Verification

#	Fidelity Indicator	Fully Observed	Partially Observed	Not Observed	N/A	Observer Notes
E1	Condition operated within its own separate LMS environment	☐	☐	☐	☐	
E2	Platform/tool access was restricted to the assigned condition (no cross-condition access observed)	☐	☐	☐	☐	
E3	AI activity logs (where applicable) were available and consistent with the assigned condition's protocol	☐	☐	☐	☐	
E4	No evidence of cross-condition material sharing, task exposure, or instructor cross-over was observed	☐	☐	☐	☐	

Section F. Adherence Scoring Summary

**Total indicators applicable to this session (N)**	
**Number rated "Fully Observed"**	
**Number rated "Partially Observed"**	
**Number rated "Not Observed"**	
**Adherence % = (Fully Observed / N) × 100**	
**Fidelity threshold met? (≥ 90% adherence)**	

Each applicable fidelity indicator should be rated as Fully Observed, Partially Observed, Not Observed, or Not Applicable (N/A). Session adherence is calculated as the percentage of applicable indicators rated Fully Observed: Adherence (%) = (Number of Fully Observed

Indicators ÷ Total Applicable Indicators) × 100. Sessions achieving ≥ 90% adherence are considered to have met the predefined treatment-fidelity criterion. When two observers independently evaluate the same session, inter-rater agreement should be estimated using Cohen's κ.

Observer Certification

Observer Signature		Date Completed	

S3. Appendix A — Technical Architecture and Adaptive Decision Rules of the AI-Adaptive Reading System

Table S3.1 Architecture and Functional Modules

Layer	Module	Primary Function	Inputs	Outputs
L1	Learner Analytics Engine	Computes learner-state metrics in real time	Responses, timing, navigation logs	Accuracy, latency, error indices
L2	Cognitive Alignment Module	Estimates learner–task fit	Metrics from L1	Alignment score
L3	Rule-Based Adaptation Engine	Applies deterministic thresholds	Alignment + CEFR tier	Adaptation commands
L4	Instructional Generator	Modifies texts, tasks, and scaffolds	Adaptation commands	Personalized materials
L5	Logging & Audit Layer	Stores all system decisions	All layers	Replication files

Table S3.2 Learner-State Indicators and Operational Definitions

Indicator	Computation	Rolling Window	Cognitive Interpretation
Accuracy Rate	Correct/total responses	5 items	Mastery vs overload
Response Latency	Mean seconds per item	5 items	Processing demand
Error Pattern Index	Lexical/inferential/discourse errors	Session	Skill deficit
Help Requests	Hint activations	Session	Dependency
Backtracking Events	Navigation reversals	Session	Monitoring effort
Self-Reported Difficulty	1–5 scale	Session	Subjective load

Table S3.3 Adaptable Instructional Dimensions

Dimension	Adjustable Features	Purpose
Text Complexity	Lexical bands, clause depth, cohesion markers	Control intrinsic load
Lexical Support	Glosses, L1 hints, examples	Reduce decoding burden
Scaffolding Intensity	Prompts, outlines, and previews	Support comprehension
Task Difficulty	Literal → inferential → evaluative	Promote depth
Feedback Specificity	Binary → explanatory → strategic	Build metacognition

Table S3.4 Threshold Rules for Real-Time Adaptation

Trigger Condition	Threshold	Action	Cognitive Rationale
Sustained high accuracy	≥ .80 for 8 items	Increase tier	Under-challenge
Sustained low accuracy	≤ .60 for 5 items	Decrease tier	Overload
Slow responses	+1 SD from baseline	Add scaffolding	Processing strain
Frequent lexical errors	>40% lexical	Increase glossing	Vocabulary gap
Inferential errors	>40% inferential	Reduce cohesion demands	Discourse overload
Low help + high accuracy	<10% + ≥.80	Fade scaffolds	Independence
High help	>30%	Maintain tier	Avoid oscillation

Table S3.5 Parameter Ranges and Stability Constraints

Parameter	Lower Bound	Upper Bound	Constraint
Lexical frequency	2,000 families	8,000 families	CEFR aligned
Sentence length	12 words	28 words	Syntax ceiling
Inferential prompts	1 / paragraph	4 / paragraph	Avoid overload
Gloss density	1/40 words	1/10 words	Fade gradually
CEFR shift	–1 band	+1 band	No jumps
Session change	0.5 SD	1 SD	Anti-oscillation

S4. Appendix C — Parallel-Form Assessment Blueprint and Comparability Framework

Table S4.1 (Table C1). Parallel-Form Assessment Blueprint and Comparability Framework

Component	Pre-Test Form	Post-Test Form	Comparability Control
Number of passages	3	3	Matched structure
Total items	40	40	Equivalent item load
Mean passage length	≈890 words	≈905 words	Comparable text length
CEFR target range	A2–B2	A2–B2	Same proficiency coverage
Primary genres	Informational/academic	Informational/academic	Genre-matched
Lexical profile	CEFR-aligned academic vocabulary	CEFR-aligned academic vocabulary	Comparable lexical coverage
Syntactic complexity	Moderate clause density; mixed sentence structures	Moderate clause density; mixed sentence structures	Comparable syntactic complexity
Literal retrieval items	14	13	Balanced domain coverage
Local inference items	8	9	Comparable inferential demand
Global inference items	7	7	Comparable higher-order inference demands
Vocabulary-in-context items	6	6	Equivalent lexical reasoning demands
Anchor items for equating	12	12	Common-item Rasch equating
Item difficulty range	−1.72 to +1.81 logits	−1.69 to +1.84 logits	Difficulty alignment

Expert review	2 EAP specialists + 1 IELTS-trained applied linguist	Same	Content-validation consistency
Sample task types	Multiple choice, matching, inference, vocabulary interpretation	Same	Format equivalence

S5. The 18-Item Learner-Experience Questionnaire: Full Item Pool, Administration, and Scoring

This section presents the complete, finalized 18-item Learner-Experience Questionnaire referenced in Section 3.7.4 of the main text and validated via confirmatory factor analysis (see Table S6.1–S6.3 below). The instrument comprises four theoretically defined constructs Cognitive Load (5 items), Engagement (5 items), Instructional Comfort (4 items), and perceived ownership of reading achievement(4 items) developed through adaptation of established educational-psychology and computer-assisted language learning measures and reviewed by three content experts (two EAP specialists and one applied-linguistics/educational-measurement specialist) for relevance, clarity, and contextual appropriateness for tertiary EFL learners. Minor wording revisions were made following expert review prior to pilot testing (Spearman's $r = .878$, $p = .001$, two-week test–retest interval).

Response Format and Administration

All items are rated on a five-point Likert scale: 1 = Strongly Disagree, 2 = Disagree, 3 = Neither Agree nor Disagree, 4 = Agree, 5 = Strongly Agree. The questionnaire was administered once, immediately following the 12-week intervention, under standardized classroom conditions and completed anonymously. Items are presented to respondents in randomized order within each construct block to reduce order effects; the ordering below follows the construct structure for clarity of presentation. Standardized factor loadings from the four-factor confirmatory factor analysis ($N = 87$; see Table S5.2 in the CFA section of this document) are reported alongside each item for reference.

Item Pool

Code	Item Wording	Standardized Loading
**Cognitive Load (5 items)**		

CL1	The reading tasks required a great deal of mental effort.	*.78*
CL2	I often felt rushed to keep up with the pace of the reading tasks.	*.82*
CL3	I had to concentrate very hard to understand the reading materials.	*.69*
CL4	I frequently felt that there was not enough time to complete the tasks.	*.74*
CL5	I found it difficult to process all the information in the reading tasks at once.	*.71*
**Engagement (5 items)**		
E1	I was fully focused on the reading activities during the sessions.	*.80*
E2	I actively participated in the reading tasks.	*.77*
E3	I found the reading activities interesting and enjoyable.	*.85*
E4	I looked forward to the reading sessions.	*.68*
E5	I put a great deal of effort into understanding the reading materials.	*.73*
**Instructional Comfort (4 items)**		
IC1	I felt comfortable with the way the reading instruction was delivered.	*.76*
IC2	I felt confident about what was expected of me during the reading tasks.	*.81*
IC3	The instructional approach made me feel calm rather than anxious.	*.70*
IC4	I understood clearly how to complete each reading task.	*.79*
**perceived ownership of reading achievement(4 items)**		
AI1	I felt that my understanding of the texts was the result of my own effort.	*.83*
AI2	I felt a genuine sense of ownership over my reading achievements.	*.77*
AI3	I felt that the support I received did not replace my own thinking.	*.72*
AI4	I felt that my success in understanding the texts reflected my own ability.	*.80*

Scoring

Each subscale is scored as the unweighted mean of its constituent items (after reverse-scoring, if any; no items in this instrument require reverse-scoring, as all items were worded in the direction of their respective construct). Higher Cognitive Load scores indicate greater perceived cognitive burden; higher scores on Engagement, Instructional Comfort, and perceived ownership of reading achievement indicate more favorable learner-experience perceptions. Subscale scores range from 1 to 5. Items CL2 and CL4 (both referencing perceived time pressure during reading) share a theoretically justified correlated residual in the measurement model (modification index = 14.2; $\Delta\chi^2 = 12.8$, $\Delta df = 1$, $p < .001$); this does

not affect scoring procedures but should be retained in any re-estimated CFA or SEM models using this instrument.

Psychometric Summary

Internal consistency (Cronbach's α) was acceptable to excellent across subscales: Cognitive Load α = .88 (item-total range .69–.82), Engagement α = .86 (.68–.85), Instructional Comfort α = .81 (.70–.81), and perceived ownership of reading achievementα = .90 (.72–.83). Full confirmatory factor analysis results, including overall model fit indices, the complete standardized factor loading matrix, and latent factor correlations, are reported in Section S6 below.

S6. Confirmatory Factor Analysis (CFA) of the Learner-Experience Questionnaire

A four-factor confirmatory factor analysis (CFA) was conducted to evaluate the internal structure of the 18-item learner-experience questionnaire. The hypothesized measurement model specified four correlated latent constructs: Cognitive Load (5 items), Engagement (5 items), Instructional Comfort (4 items), and perceived ownership of reading achievement(4 items), with each item loading only on its designated latent factor. Because responses were collected using five-point Likert scales, the model was estimated using the weighted least squares mean- and variance-adjusted (WLSMV) estimator implemented in the *lavaan* package (version 0.6-18) in R version 4.4.1. Theta parameterization was used, latent factor variances were fixed to one for identification, and all standardized factor loadings were freely estimated. Missing data were minimal (<2% of item responses) and were handled using the default WLSMV estimation procedure; consequently, all participants (N = 87) were retained in the analysis.

Table S6.1. Overall Confirmatory Factor Analysis Model Fit

Fit Index	Value
χ^2(df)	187.42 (129)
χ^2/df	1.45
Comparative Fit Index (CFI)	.963
Tucker-Lewis Index (TLI)	.955
Root Mean Square Error of Approximation (RMSEA)	.071
90% CI for RMSEA	[.048, .092]
Standardized Root Mean Square Residual (SRMR)	.061
Sample size	87
Estimator	WLSMV
Software	R 4.4.1; *lavaan* v0.6-18

Table S6.2. Standardized Factor Loadings

Item	Latent Construct	Loading
**CL1**	Cognitive Load	.78
**CL2**	Cognitive Load	.82
**CL3**	Cognitive Load	.69
**CL4**	Cognitive Load	.74
**CL5**	Cognitive Load	.71
**E1**	Engagement	.80
**E2**	Engagement	.77
**E3**	Engagement	.85
**E4**	Engagement	.68
**E5**	Engagement	.73
**IC1**	Instructional Comfort	.76
**IC2**	Instructional Comfort	.81
**IC3**	Instructional Comfort	.70
**IC4**	Instructional Comfort	.79
**AI1**	Perceived ownership of reading achievement	.83
**AI2**	perceived ownership of reading achievement	.77
**AI3**	perceived ownership of reading achievement	.72
**AI4**	perceived ownership of reading achievement	.80

Note. All standardized factor loadings were statistically significant ($p < .001$).

Table S6.3. Latent Factor Correlations

Factor	Cognitive Load	Engagement	Instructional Comfort	perceived ownership of reading achievement
**Cognitive Load**	1.00			
**Engagement**	−.42	1.00		
**Instructional Comfort**	−.55	.61	1.00	
**perceived ownership of reading achievement**	−.31	.58	.49	1.00

The hypothesized four-factor model demonstrated acceptable-to-good overall fit (Table S6.1). Standardized factor loadings ranged from .68 to .85 (Table S6.2), and all loadings were statistically significant ($p < .001$), indicating substantial relationships between the observed indicators and their intended latent constructs. Correlations among the latent variables were moderate in magnitude (Table S6.3) and followed theoretically expected directions. Cognitive Load showed moderate negative associations with Engagement, Instructional Comfort, and perceived ownership of reading achievement, whereas Engagement was positively associated with both Instructional Comfort and perceived ownership of reading achievement. These findings support the structural validity of the questionnaire while indicating that the four constructs are related but empirically distinguishable.

Inspection of modification indices identified one theoretically defensible localized area of model strain involving the residual covariance between items CL2 and CL4 (Modification Index = 14.2). Because both items refer to perceived time pressure during reading, a correlated residual was specified to account for shared wording-related variance rather than additional latent construct variance. This theoretically justified modification significantly improved model fit ($\Delta\chi^2 = 12.8$, $\Delta df = 1$, $p < .001$) without altering the substantive four-factor structure. No further

post hoc modifications were introduced because all remaining modification indices were below the predefined threshold (MI < 10) or lacked adequate theoretical justification.

S7. Exploratory Sensitivity and Robustness Analyses

Table S7.1 Sensitivity Analyses Across Alternative Specifications

Model Specification	Time × AI β	p
Mixed-effects (primary)	0.44	.002
Without a class random effect	0.46	.001
Raw scores	0.41	.003
ANCOVA	0.39	.005

Table S7.2 Pairwise Effect Sizes for Reading Gains

Comparison	Cohen's d	95% CI	Magnitude
AI vs Control	0.69	0.16, 1.22	Medium–large
Teacher vs Control	0.52	0.01, 1.03	Medium
AI vs Teacher	0.22	−0.30, 0.73	Small

S8. Representative Qualitative Quotations Supporting Thematic Analysis

Table S8.1 Thematic Patterns in Learner Reflections Across Instructional Conditions

Theme	Representative Quotation
Autonomy Through Adaptive Clarity	“The system adjusted when I struggled, but I still felt the answers came from my understanding.” (AI-Adaptive learner)
Reduced Cognitive Pressure	“The reading became easier step by step, so I didn’t panic when texts became difficult.” (AI-Adaptive learner)
Guided but Teacher-Dependent Confidence	“The teacher explained things clearly, but I still waited for help before moving on.” (Teacher-Differentiated learner)
Passive Task Compliance	“Sometimes the reading felt too difficult, and I just followed the class without really understanding.” (Control learner)
Preserved Ownership of Achievement	“Even with support, I felt the improvement was mine because I was still doing the reading myself.” (AI-Adaptive learner)
Predictability as Psychological Safety	“I liked that the tasks matched my level instead of suddenly becoming confusing.” (AI-Adaptive learner)
