# Supplementary material for: Personalization and the Homogenization Debate in AI-Adaptive EFL Reading: Cognitive Load, Learner Agency, and Reading Development
Source: Behav Sci (Basel). 2026 Aug 25;16(9):1478. doi: 10.3390/bs16091478 (PMC13603526; doi:10.3390/bs16091478)
Supplement: Supplementary file 1 [file behavsci-16-01478-s001.zip › Supplementary File S0.pdf]

بسم الله الرحمن الرحيم

Kingdom of Saudi Arabia
Ministry of Education
Imam Mohammad Ibn Saud
Islamic University
College of Languages & Transalation

VISION 2030 رؤية
المملكة العربية السعودية
KINGDOM OF SAUDI ARABIA

المملكة العربية السعودية
وزارة التعليم
جامعة الإمام محمد بن سعود الإسلامية
كلية اللغات والترجمة

Subject: Approval of Research Application in Arts and Humanities

This letter is to inform you that the Ethical Board Committee within the College of Languages and Translation at Imam Mohammad Ibn Saud Islamic University has examined and approved the research application submitted by Dr. Talal Alghizzi and Ms. Latifah Alghamdi on May 15, 2024, under reference number [1037]. The title of the approved research is "*The Persolnlization-Homoginization Paradox: How AI Reshapes Cognitive Load, Identity, and Learners Pathways in EFL Reading*".

The committee carefully reviewed the research proposal and found it to be of significant value and relevance to the arts and humanities domain. The proposed research demonstrates a clear objective, sound methodology, and potential contributions to the existing body of knowledge in the field. We are confident that this research project holds immense potential for advancing understanding and fostering intellectual growth in the arts and humanities.

The research proposal successfully passed the rigorous ethical review process conducted by our committee, ensuring adherence to the highest ethical standards in research. The committee was satisfied with the measures outlined by the researchers to protect the rights, privacy, and well-being of participants, as well as the appropriate handling of data and compliance with relevant regulations.

We hereby approve to proceed with the proposed research project, subject to the researcher's continued adherence to the ethical guidelines outlined by our College and any additional requirements or conditions specified by the Ethical Board Committee.

Ethical Board Committee

Head of the Department of English Language and Literature

**Dr. Mohammad Alhammad**
